# New Dromaeosaurids (Dinosauria: Theropoda) from the Lower Cretaceous of Utah, and the Evolution of the Dromaeosaurid Tail

**DOI:** 10.1371/journal.pone.0036790

**Published:** 2012-05-15

**Authors:** Phil Senter, James I. Kirkland, Donald D. DeBlieux, Scott Madsen, Natalie Toth

**Affiliations:** 1 Department of Biological Sciences, Fayetteville State University, Fayetteville, North Carolina, United States of America; 2 Utah Geological Survey, Salt Lake City, Utah, United States of America; 3 Rio Tinto Center, Utah Museum of Natural History, Salt Lake City, Utah, United States of America; University of Pennsylvania, United States of America

## Abstract

**Background:**

The Yellow Cat Member of the Cedar Mountain Formation (Early Cretaceous, Barremian? – Aptian) of Utah has yielded a rich theropod fauna, including the coelurosaur *Nedcolbertia justinhofmanni*, the therizinosauroid *Falcarius utahensis*, the troodontid *Geminiraptor suarezarum*, and the dromaeosaurid *Utahraptor ostrommaysorum*. Recent excavation has uncovered three new dromaeosaurid specimens. One specimen, which we designate the holotype of the new genus and species *Yurgovuchia doellingi*, is represented by a partial axial skeleton and a partial left pubis. A second specimen consists of a right pubis and a possibly associated radius. The third specimen consists of a tail skeleton that is unique among known Cedar Mountain dromaeosaurids.

**Methodology/Principal Findings:**

*Y. doellingi* resembles *Utahraptor ostrommaysorum* in that its caudal prezygapophyses are elongated but not to the degree present in most dromaeosaurids. The specimen represented by the right pubis exhibits a pronounced pubic tubercle, a velociraptorine trait that is absent in *Y. doellingi*. The specimen represented by the tail skeleton exhibits the extreme elongation of the caudal prezygapophyses that is typical of most dromaeosaurids. Here we perform a phylogenetic analysis to determine the phylogenetic position of *Y. doellingi*. Using the resulting phylogeny as a framework, we trace changes in character states of the tail across Coelurosauria to elucidate the evolution of the dromaeosaurid tail.

**Conclusions/Significance:**

The new specimens add to the known diversity of Dromaeosauridae and to the known diversity within the Yellow Cat paleofauna. Phylogenetic analysis places *Y. doellingi* in a clade with *Utahraptor*, *Achillobator*, and *Dromaeosaurus*. Character state distribution indicates that the presence of intermediate-length caudal prezygapophyses in that clade is not an evolutionarily precursor to extreme prezygapophyseal elongation but represents a secondary shortening of caudal prezygapophyses. It appears to represent part of a trend within Dromaeosauridae that couples an increase in tail flexibility with increasing size.

## Introduction

A diversity of theropods is found in the fauna of the Cedar Mountain Formation (Lower Cretaceous) of Utah. A large carnosaur similar to *Acrocanthosaurus* is known from fragmentary material from the Ruby Ranch Member [1]. The Yellow Cat Member has yielded the coelurosaur *Nedcolbertia justinhofmanni* [2], the therizinosauroid *Falcarius utahensis* [3], the troodontid *Geminiraptor suarezarum* [4], and the large dromaeosaurid *Utahraptor ostrommaysorum* [5]. Here we describe three new theropod specimens from the Yellow Cat Member, all of which are members of Dromaeosauridae. One specimen, UMNH (Natural History Museum of Utah, Salt Lake City, Utah, United States of America) VP 20211, includes several vertebrae and part of a pubis; we designate this specimen the holotype of a new genus and species: *Yurgovuchia doellingi*. Another specimen is represented by a pubis (UMNH VP 21752) and possibly also by a radius (UMNH VP 21751) found near it. The third specimen (UMNH VP 20209) is a tail skeleton.

Dromaeosauridae is a diverse family of predatory dinosaurs with a plethora of species that have been discovered within the last two decades [5]–[26] and a few that were known previously [27]–[31]. The family is known from the Lower and Upper Cretaceous of North and South America [5], [6], [11], [16], [19], [24], [27], [29], [30] and Asia [10], [12], [14], [15], [17], [18], [20]–[22], [25], [28], [31] and from the Upper Cretaceous of Europe [8], [13], Africa [32], and Madagascar [7]. A wide range of body sizes is known in the family, with the smallest about the size of a mockingbird [12] and the largest about the size of an emu [5]. Dromaeosaurids are remarkable for the presence of an enlarged, recurved claw on the second toe that may have functioned as a predatory weapon [33], a weapon for intraspecific aggression [34], a climbing aid [35], a digging tool [34], or a combination of functions. Specimens are known that are covered in birdlike feathers [14], [15], and because of their close relationship with birds [19], [21], [36] dromaeosaurids are important in studies of the origin of avian flight [15], [37].

In most members of the family the centra of the tail are encased in a bundle of bony rods that lie parallel to each other across the lengths of several centra, binding the tail into a rod [18], [29], [38], [39]. These bony rods consist of bifurcating prezygapophyses and bifurcating left and right cranial processes of hemal arches. It is hypothesized that this bundle of rods increased tail stiffness and that this is functionally related to the use of the tail as a dynamic stabilizer [29]. The term “caudotheca,” from the Latin *cauda* (tail) and *theca* (case, covering), was recently introduced [36] for the sheath of elongated processes from prezygapophyses and chevrons that encase the tail. The term is not taxon-specific and can be used in reference to the similar structure that is present in basal pterosaurs [40].

There are exceptions to the presence of the caudotheca in Dromaeosauridae. It is absent in the basal dromaeosaurid subfamily Unenlagiinae [7], [19]. An intermediate state is present in the large, closely related dromaeosaurids *Achillobator* and *Utahraptor*. In both cases, distal caudal vertebrae have elongated prezygapophyses, but the prezygapophyses are not known to extend much farther than the length of one caudal centrum [5], [9]. Here, we introduce the term “hemicaudotheca” for this condition.

To facilitate understanding of the results of this study, we must address the definitions of three relevant terms: Microraptoria, Eudromaeosauria, and transition point. The name Microraptoria was introduced in 2004 for the clade of dromaeosaurids more closely related to *Microraptor* than to *Velociraptor* or *Dromaeosaurus* [41]. In some subsequent studies the clade has been called Microraptorinae [19], [21], [24], [36], but that name is a junior synonym of Microraptoria. The name Eudromaeosauria was recently introduced as the least inclusive clade containing *Saurornitholestes langstoni*, *Deinonychus antirrhopus*, *Dromaeosaurus albertensis*, and *Velociraptor mongoliensis* [24]. This clade is the sister taxon to Microraptoria [19], [24], [36].

Russell introduced the term “transition point” in 1972 in reference to ornithomimid tails [42], but the term has since become routinely applied to dromaeosaurids and other theropods. Russell defined the term as the point in the tail “between the last vertebra bearing transverse processes and the first with distinctly elongate prezygapophyses” and made it clear that the term was meant to refer to a point of abrupt change in vertebral morphology that divides the tail into two distinct segments [42]. The term is occasionally used in reference to theropods in which the caudal series does not exhibit two distinct, abruptly divided segments [43]–[45], but if the term is used as originally intended it is inapplicable to such taxa. Among theropods, abrupt division of the tail into two distinct segments is present only in Ornithomimidae and Paraves. In the former, the division is marked not only by loss of transverse processes and the elongation of prezygapophyses but also by the gain of an cranial process on each hemal arch so that the hemal arches are shaped like an inverted T [46], [47]. The term “type 1 transition point” was recently introduced for this condition [36]. In Paraves the division is marked by loss of transverse processes, extreme reduction in neural spines, elongation of caudal centra, and the gain of an inverted T shape in the hemal arches, but not by prezygapophyseal elongation [7], [48]–[51]. The term “type 2 transition point” was recently introduced for this condition [36]. In contrast, along the caudal series in other coelurosaurs (Tyrannosauroidea, Compsognathidae, Therizinosauroidea, and Oviraptorosauria), changes in morphology (reduction in transverse process and neural spine size, prezygapophyseal elongation, and hemal arch shape change) are gradual, so that no distinct division of the tail into two segments is present [45], [52]–[58]. The term should therefore not be used in such cases.

### Geological Setting

**Figure 1:**
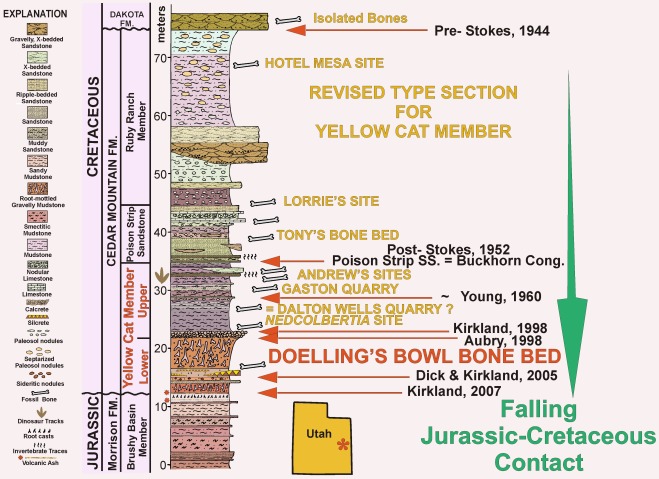
Revised stratigraphic section for type area of Yellow Cat and Poison Strip Members of the Cedar Mountain Formation along the east side of the Yellow Cat Rd. south of Exit 193 on Interstate 70. Arrows indicate the placement of the Jurassic-Cretaceous boundary by previous authors. Tan writing indicates the relative stratigraphic position of a number of important dinosaur localities in the area [61], [63], [99], [100].

All three specimens described here are from the Yellow Cat Member of the Cedar Mountain Formation, which is divided into upper and lower parts (Fig. 1). The upper Yellow Cat Member has been dated as about 124 Ma (Lower Cretaceous: Aptian) based on detrital zircons [59]. This is close to the cutoff between the Barremian Stage and the Aptian Stage [60], suggesting that the lower Yellow Cat Member may be Barremian in age. Both the upper and lower Yellow Cat have recently yielded several significant fossil specimens [3], [4], [6]–[63].

**Figure 2:**
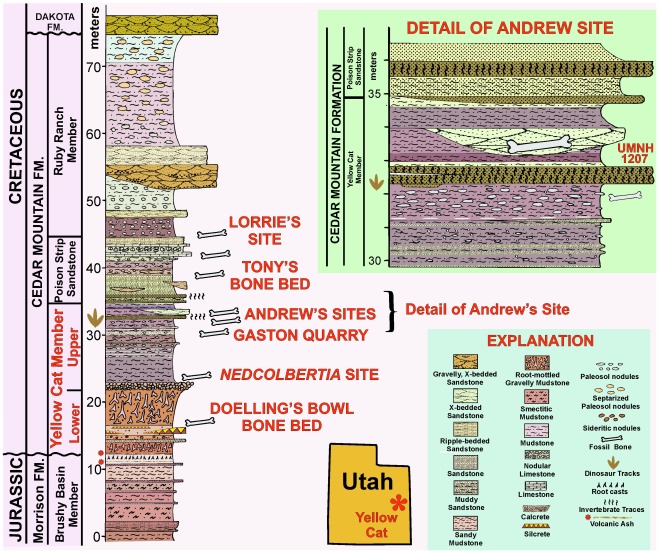
Detailed stratigraphic correlation of the lower Yellow Cat Member of the Cedar Mountain Formation at its type section with the stratigraphic section at Doelling’s Bowl five kilometers to the east, showing the stratigraphic position of the Doelling’s Bowl dinosaur bone bed.

**Figure 3:**
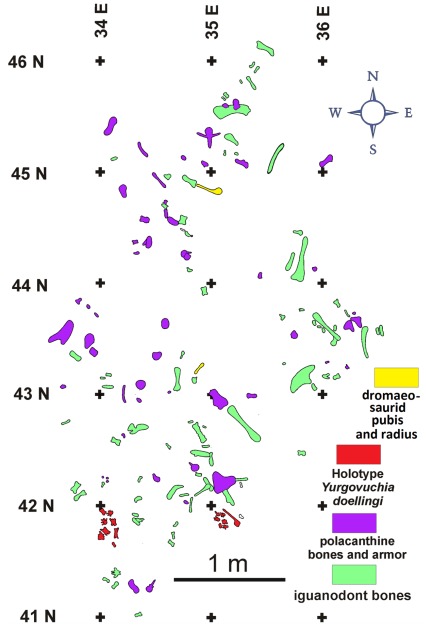
A small portion of the Doelling’s Bowl dinosaur bone bed at Don’s Place showing the disposition of bones assigned to the type specimen of *Yurgovuchia doellingi*.

The holotype of *Yurgovuchia doellingi* and the isolated pubis (UMNH VP 21752) and radius (UMNH VP 21751) are from a portion of the Doelling’s Bowl bone bed (Gr 300v) that is designated as Don’s Place (Fig. 2, 3). Initial discovery of the site occurred in 2005 when Don DeBlieux found the vertebrae and associated pubis of *Y. doellingi*, while Scott Madsen was simultaneously investigating some large skeletal elements a few tens of meters to the north and Jim Kirkland was excavating a polacanthine ankylosaur spine at his initial 1991 tooth and scute locality [63], a site approximately two hundred meters to the west. All of these sites were later found to be portions of the same extensive bone bed.

The identification of the stratigraphic position of the Doelling’s Bowl bone bed necessitated a reevaluation of the stratigraphic position of the Jurassic-Cretaceous boundary and the base of the Cedar Mountain Formation in this area [63]. For a number of years a widespread multistoried paleosol (calcrete) had been used to define the base of the Cedar Mountain Formation [61], [64]–[67]. That marker bed is now placed in the middle of the Yellow Cat Member of the Cedar Mountain Formation and is used to split the Yellow Cat into a lower and upper part in its type area along the north side of Arches National Park (Fig. 1). The lithology and fauna of the lower Yellow Cat are typical of the Cedar Mountain formation and differ markedly from the underlying Brushy Basin Member of the Morrison Formation [59], [63]. There is no radiometric date available yet for the lower Yellow Cat.

The lower Yellow Cat Member is characterized by mottled paleosols with matrix-supported chert pebbles. Its lower contact is placed at the first occurrence of matrix-supported chert pebbles above smectitic mudstones characteristic of the Upper Jurassic Brushy Basin Member of the Morrison Formation and is often characterized by iron staining and iron-rich nodules. The Doelling’s Bowl Dinosaur bone bed is centimeters to decimeters above an interval of distinctive chert layers that form a local marker “zone” in this area around Arches National Park [63].

Common in the Doelling’s Bowl dinosaur bone bed and the underlying chert interval are silicified, horizontally oriented, winding roots, whose rarely preserved internal structure is similar to that of the “pseudo-trunk” of the Early Cretaceous aberrant fern *Tempskya* [68], [69], [70]. The presence of these horizontally oriented, “fern-like,” root traces suggests that the bone bed represents a wet environment. Within the bone bed, these root traces have been found to be most abundant in the Don’s Place area, where some of the root traces followed along the surfaces of bones suggesting that these roots were leaching nutrients out of the bones.

The Doelling’s Bowl dinosaur bone bed extends over tens of acres with mostly scattered teeth, ankylosaur ossicles, and isolated bones in the western exposures and better preserved and much more abundant skeletal remains in the eastern exposures. The preserved dinosaurs are dominated by iguanodonts (at least ten individuals) and polacanthine ankylosaurs (at least three individuals) [60]. Other identified dinosaur remains include the type specimen of *Y. doellingi*, one or possibly two associated sauropod skeletons, a hypsilophodont-grade ornithopod jaw, and teeth from a large carnosaur. Additionally, teeth and a few crocodilian bones have been recognized.

The bone bed is a relatively low diversity, multitaxic bone bed of associated, if not articulated, skeletons, and while there is some orientation of long bones the close association of elements from the same individuals suggests low energy [70]. The distribution of skeletal elements (Fig. 3), together with the iron-rich, “hydromorphic gley soils” suggest a wet, perhaps boggy, environment for the Doelling’s Bowl dinosaur bone bed, unlike the drier settings interpreted for many other Cedar Mountain dinosaur localities.

**Figure 4:**
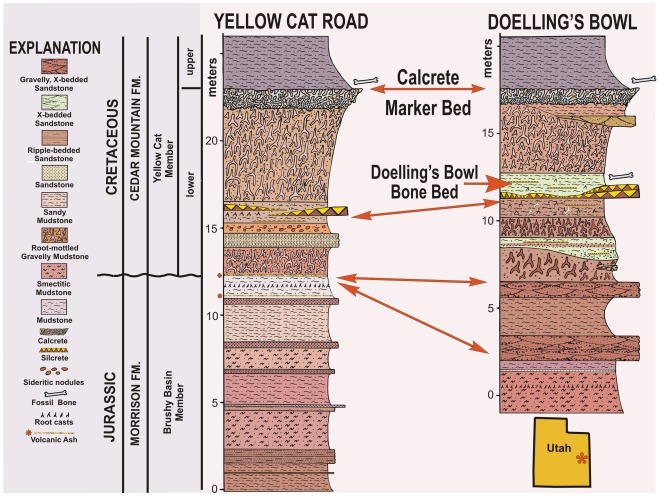
Stratigraphic data for Andrew’s Site. Stratigraphic section of the Cedar Mountain Formation in the type area of the Yellow Cat Member [61], with major dinosaur localities [62], [63] in area noted and detail of section spanning the locality (Andrew’s Site, Gr v290) from which the dromaeosaur tail was collected.

**Figure 5:**
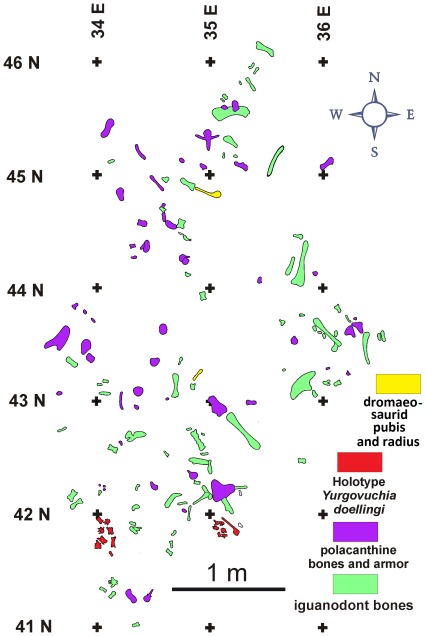
Quarry map of Andrew’s Site.

The new dromaeosaurid tail skeleton (UMNH VP 20209) is from Andrew’s Site (Gr 290v), a small but highly significant dinosaur site near the top of the Yellow Cat Member of the Cedar Mountain Formation (Fig. 4, 5). The site has yielded several significant fossil specimens. These include the skulls of an iguanodont, a terrestrial crocodilian, and a new species of mammal [61]–[63].

## Methods

### Phylogenetic Analysis

For this analysis we used the phylogenetic data matrix of a recent study [36], with the following improvements. Two new characters (characters 301 and 307) relating to the pelvic girdle have been added, and character 306 (pubic orientation) has been re-coded for some taxa according to new insights published here in the Discussion. The *Scipionyx* Operational Taxonomic Unit (OTU) and other compsognathid OTUs have been updated with new information from a recent study [71]. The *Chirostenotes* OTU has been updated by removal of data that came from the holotype of the newly recognized genus *Epichirostenotes* [72], a specimen that was originally described as a specimen of *Chirostenotes* [73]. The *Ornithomimus* OTU has been updated with information from a recent study [74]. In that study, a small opening ventral to the maxillary fenestra was called the promaxillary fenestra [74]. However, the absence of that opening in basal ornithomimosaurs suggests that its appearance in advanced ornithomimosaurs is neomorphic. Therefore, the phylogenetic data matrix used here does not recognize the presence of the promaxillary fenestra in *Ornithomimus* and other advanced ornithomimosaurs.

The data matrix for the phylogenetic analysis is given in Appendix S1, and the character list is given in Appendix S2.

Phylogenetic analysis was performed with PAUP 4.0 for Windows [75]. A heuristic search with 1000 random addition-sequence replicates was performed, with no limit to “maxtrees.” The decay index (Bremer support) of each clade was found with the same software after insertion of a command line that was created using the program MacClade 3.08a [76]. The analysis was performed a second time after deletion of several deinonychosaurian taxa known only from fragmentary material, to see whether this would influence tree topology or statistics.

### Tail Evolution Scenario

We used specimens and literature to compare caudal character states across Coelurosauria for taxa in which articulated tail skeletons are known or for which enough of the caudal series is known to be able to deduce the relevant character states (Table 1). We then used these data to create a scenario for the evolution of the dromaeosaurid tail, using as a phylogenetic framework the results of the phylogenetic analysis performed here.

**Table 1:** Data from tails of coelurosaurian theropods.

Taxon and references	NC	TP	E	PZ	C	2×	TrPt
Tyrannosauridae:							
*Gorgosaurus libratus* [52]	31 (+4)	13	−	−	−	No	No
*Tyrannosaurus rex* [56]	>34	16	−	−	−	No	No
Compsognathidae:							
*Huaxiagnathus orientalis* [45]	>25	17	−	−	−	No	No
*Sinocalliopteryx gigas* [58]	49	16	−	−	−	No	No
*Sinosauropteryx prima* [54]	>64	17	−	−	−	No	No
Ornithomimosauria:							
*Gallimimus bullatus* [41]	36–39	14	−	−	−	No	Yes
*Harpymimus okladnikovi* [95]	>34	12	−	−	−	No	Yes
*Shenzhousaurus orientalis* [47]	>16	11	−	−	−	No	Yes
Therizinosauroidea:							
*Alxasaurus elesitaiensis* [96]	>19	13	−	−	−	No	No
*Beipiaosaurus inexpectus* [57]	30	?	−	−	−	No	No
*Neimongosaurus yangi* [55]	22 (+3 to 8)	>12	−	−	−	No	No
*Nothronychus graffami* [97]	23 (+3)	12	−	−	−	No	No
Oviraptorosauria:							
*Caudipteryx* sp. (IVPP V 12430)	9	−	−	−	−	No	No
*Khaan mckennai* (IGM 100/1127)	26 (+2)	21	−	−	−	No	No
*Nomingia gobiensis* [53]	24	18	−	−	−	No	No
Avialae							
*Archaeopteryx* sp. [49]	22	5	6	−	−	Yes	Yes
*Epidendrosaurus ningchengensis* [50]	22 (+5?)	2	?	−	−	Yes	Yes
*Jeholornis prima* [51]	24–27	2	3	−	−	Yes	Yes
Troodontidae:							
*Anchiornis huxleyi* [48]	20 (+6?)	3	5	−	−	Yes	Yes
*Sinornithoides dongi* [98]	27	9	9	−	−	Yes	Yes
Dromaeosauridae (Unenlagiinae):							
*Buitreraptor gonzalezorum*	>14	5	8	−	−	Yes	Yes
*(cast of MPCA 245)*							
*Rahonavis ostromi* (cast of UA 8656)	>13	6	6	−	−	Yes	Yes
Dromaeosauridae (Microraptoria):							
*Cryptovolans pauli* [14]	28–20	?	5	?	4	Yes	Yes
*Microraptor gui* [15]	approx. 26	?	4–6	?	?	Yes	Yes
*Microraptor zhaoianus* [12]	24–26	6	6	6	3	Yes	Yes
*Tianyraptor ostromi* [25]	>25	?	7	?	3	Yes	Yes
Dromaeosauridae (Eudromaeosauria):							
*Bambiraptor feinbergorum*	>23	6	10	9	6	Yes	Yes
(AMNH FR 30556 and [11])							
*Deinonychus antirrhopus* [29]	36 (+4)	10	9	>10	8	No	Yes
*Tsaagan* sp. [26]	>20	>12	>8	>10	6	No	Yes
*Velociraptor mongoliensis* [38], [83]	approx. 30	11	6	10	6	No	Yes
UMNH VP 20209	?	?	?	>8*	>7*	?	?
*under the assumption that at least four							
proximal caudals are missing (see text)							

2× = mid-caudal centra at least twice as long as proximal caudal centra. E = cranialmost caudal centrum with suddenly marked elongation (length/height) as compared to more proximal caudal centra. NC = number of caudal vertebrae. PZ = cranialmost caudal contributing to caudotheca. C = cranialmost caudal contacted by caudotheca. TP = distalmost caudal with transverse process as a distinct process (not just a low ridge). TrPt = Abrupt transition point present. Numbers in parentheses under “NC” indicate estimated number of additional vertebrae beyond those preserved in the specimen. Hyphens indicate inapplicability. For cases in which data were collected directly from a specimen or cast, the specimen number is given. Institutional abbreviations: AMNH = American Museum of Natural History, New York City, New York, United States of America. IGM = Mongolian Institute of Geology, Ulaan Baatar, Mongolia. IVPP = Institute of Vertebrate Paleontology and Paleoanthropology, Beijing, People’s Republic of China. MPCA = Museo Carlos Ameghino, Cipolletti, Rio Negro Province, Argentina. UA = Université d’Antananarivo, Antananarivo, Madagascar. UMNH = Utah Museum of Natural History, Salt Lake City, Utah, United States.

### Nomenclatural Acts

The electronic version of this document does not represent a published work according to the International Code of Zoological Nomenclature (ICZN), and hence the nomenclatural acts contained in the electronic version are not available under that Code from the electronic edition. Therefore, a separate edition of this document was produced by a method that assures numerous identical and durable copies, and those copies were simultaneously obtainable (from the publication date noted on the first page of this article) for the purpose of providing a public and permanent scientific record, in accordance with Article 8.1 of the Code. The separate print-only edition is available on request from PLoS by sending a request to PLoS ONE, 1160 Battery Street, Suite 100, San Francisco, CA 94111, USA along with a check for $10 (to cover printing and postage) payable to “Public Library of Science”.

In addition, this published work and the nomenclatural acts it contains have been registered in ZooBank, the proposed online registration system for the ICZN. The ZooBank LSIDs (Life Science Identifiers) can be resolved and the associated information viewed through any standard web browser by appending the LSID to the prefix “http://zoobank.org/”. The LSID for this publication is: urn:lsid:zoobank.org:pub:4D1838FD-10BB-4754-8127-9B881D33D004.

## Results

### *Yurgovuchia Doellingi*: Systematic Paleontology

**Systematic hierarchy:**

Dinosauria Owen, 1841 [77]

Saurischia Seeley, 1887 [78]

Theropoda Marsh, 1881 [79]

Coelurosauria von Huene, 1914 [80]

Dromaeosauridae Colbert and Russell, 1969 [27]

*Yurgovuchia* gen. nov.

urn:lsid:zoobank.org:act:110C8550-8718-40C6-8AA8-8A534A33535B

*Yurgovuchia doellingi* sp. nov.

urn:lsid:zoobank.org:act:8C781C52-96FF-4377-A337-82FC875DC24E

#### Holotype

The holotype specimen is UMNH VP 20211. It includes cervical, dorsal, and caudal vertebrae; and the proximal end of a left pubis.

#### Etymology

The genus name honors the Ute Tribe of northeastern Utah. It is derived from the Ute word *yurgovuch*, meaning “coyote,” a predator of similar size to *Y. doellingi* that currently inhabits the same region.

The species name honors Helmut Doelling in recognition of his 50-plus years of geological research and mapping of Utah for the Utah Geological Survey. The Doelling’s Bowl dinosaur sites were first discovered as a result of his providing taped-together color photocopies of his then-unpublished geological maps of the Arches National Park region [81], [82] to JIK in 1990.

#### Locality and horizon

The specimen comes from Don’s Place, part of the Doelling’s Bowl bone bed in Grand County, Utah (Fig. 3). The bone bed is in the lower Yellow Cat Member (Barremian?) of the Cedar Mountain Formation (Fig. 2).

#### Diagnosis

Dromaeosaurid theropod; centrum of axis with a single pneumatopore on each side; cranial end of centrum of third cervical vertebra not beveled; cervical prezygapophyses flexed; epipophyses of cervical vertebrae above postzygapophyseal facets; cervico-dorsal vertebrae with hypapophyses and without pneumatopores; cranial faces of centra of proximal caudal vertebrae round; caudal prezygapophyses elongated distal to transition point, but not over the length of a centrum. Pubis without pubic tubercle.

### *Yurgovuchia Doellingi*: Specimen Description and Comparisons

Below, specimen numbers in parentheses indicate personal observation by PS of features not recorded in the literature.

**Figure 6:**
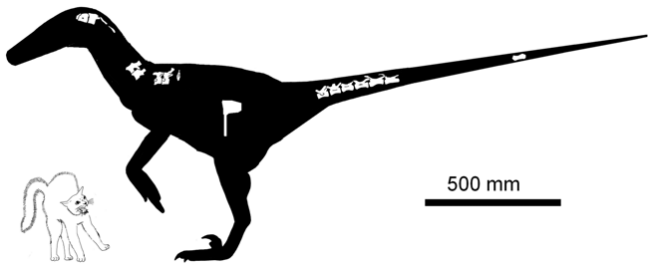
Skeletal reconstruction of *Yurgovuchia doellingi*, with anachronistic house cat to show its size.

Preserved vertebrae and parts of vertebrae include the left neural arch of the atlas, a nearly complete axis, three postaxial cervical vertebrae, partial neural arches of two more postaxial cervicals, two cervico-dorsal vertebrae, the neural arch of one dorsal vertebra, and seven caudal vertebrae (Fig 6). In all preserved vertebrae, neurocentral sutures are obliterated by coossification. This indicates that the specimen is an adult [83]. It is therefore not a juvenile specimen of *Utahraptor ostrommaysorum* but instead represents a much smaller species. Measurements are given in Table 2.

**Table 2:** Measurements of vertebrae preserved in the holotype of *Yurgovuchia doellingi*.

Figure	TH	TL	TW	CL	CHCr	CHCd	CWCr	CWCd
7A (atlantal neural arch)	13.35	12.69	27.43	−	−	−	−	−
7B (axis)	41.97	45.92	−	−	−	−	21.9	17.55
7C (cervical vertebra)	45.95*	53.65	40.29	45.1	−	−	24.35	−
7D (cervical neural arch)	−	48.6	−	−	−	−	−	−
7E (cervical neural arch)	−	5.09*	−	−	−	−	−	−
7F (cervical vertebra)	55.80*	−	69.73*	24.89*	21.27*	−	30.81*	−
7G (cervical vertebra)	46.39*	−	−	40.95*	−	19.58*	−	26.13*
7H (cervico-dorsal vertebra)	47.80*	−	−	26.42*	25.75*	23.39*	−	23.18*
7I (cervico-dorsal vertebra)	79.36*	−	−	32.04	25.96	22.73	−	22.25
7J (dorsal neural arch)	51.73*	22.90*	76.64	−	−	−	−	−
8A (caudal vertebra)	47.89*	44.63*	23.06*	40.17	22.11	27.69	24.11	22.2
8B (caudal vertebra)	45.95*	55.59*	37.32*	45.95	25.16	23.29	19.54	17.57
8C (caudal vertebra)	42.23*	58.74*	44.09*	48.03	23.35	21.64	20.78	18.54
8D (caudal vertebra)	43.2	66.92	−	52.91	22.80*	21.03*	−	16.62*
8E (caudal vertebra)	47.62*	75.81*	−	−	24.73*	23.60*	−	−
8F (caudal vertebra)	43.47*	91.32*	−	55	18.59	18.41	14.47	15.83
8G (caudal centrum)	25.07*	48.34*	−	48.34*	16.97	20.82*	19.24	−

Measurements are in mm. Asterisks indicate measurements that are correct for the vertebra in its current state but are significantly altered from the original state because relevant part(s) of the vertebra are broken, distorted, or missing. Fig.  =  figure in which vertebra is illustrated. CHCd = centrum height (caudal face). CHCr = centrum height (cranial face). CL = centrum length (as preserved). CWCd = centrum transverse width (caudal face). CWCr = centrum transverse width (cranial face). TH = total height of vertebra (or preserved part). TL = total length of vertebra (or preserved part). TW = total transverse width of vertebra (or preserved part).

**Figure 7:**
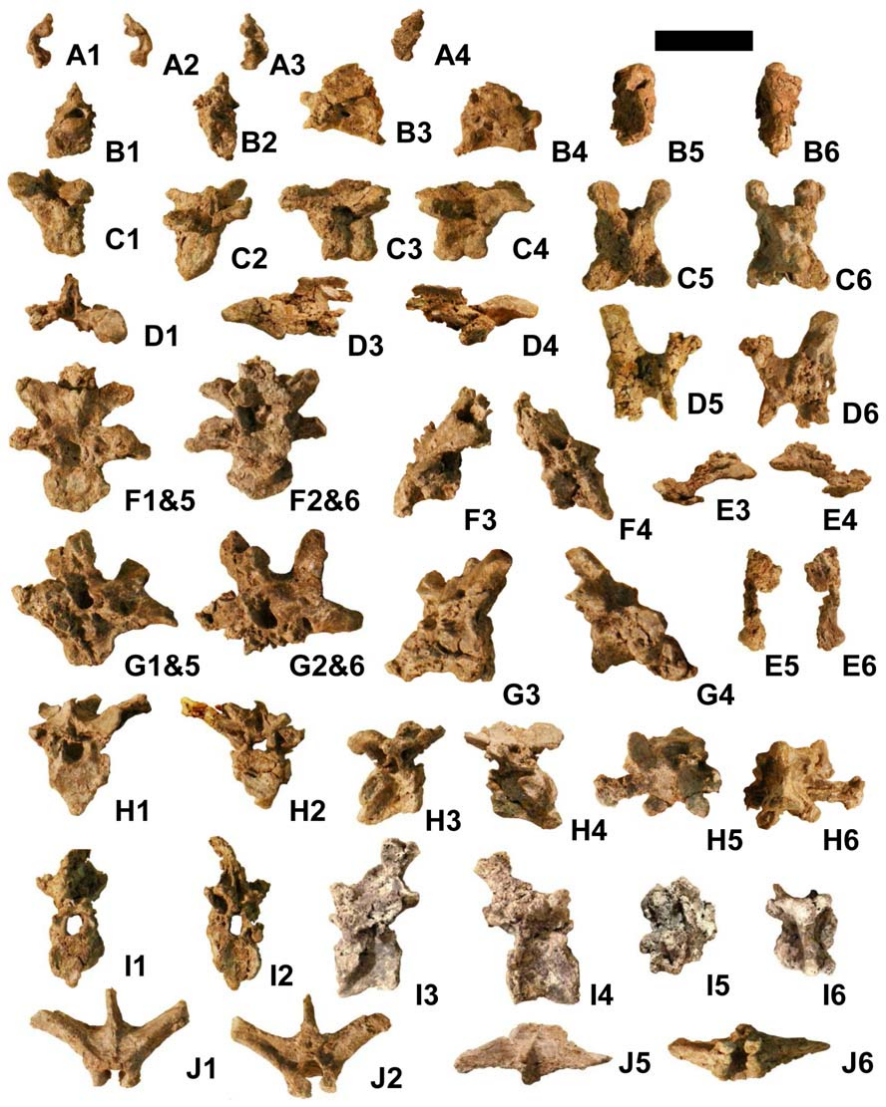
Cervical and dorsal vertebrae of *Yurgovuchia doellingi* (UMNH VP 20211). (A)–Left neural arch of atlas. (B)–Axis. (C)–Third cervical vertebra. (D)–Partial cervical neural arch. (E)–Partial cervical neural arch. (F)–Posterior cervical vertebra. (G)–Posterior cervical vertebra. (H)–Cervico-dorsal vertebra. (I)–Cervico-dorsal vertebra. (J)–Dorsal vertebral neural arch. Scale bar = 50 mm. Numbers on sub-figures refer to cranial (1), caudal (2), left (3), right (4), dorsal (5), and ventral (6) views. Such numbers joined by an ampersand indicate that cranial and dorsal (1&5) or caudal and ventral (2&6) surfaces are simultaneously visible, due to deformation. Scale bar = 50 mm.

The postzygapophysis of the atlantal neural arch is bulbous (Fig. 7A). A deep fossa is present on the medial surface between the base of the arch and the postzygapophysis. The dorsal process that extends medially toward the right neural arch is shorter and stouter than in *Deinonychus* [29].

The intercentrum and odontoid process of the axis (Fig. 7B) resemble those of *Deinonychus* [29] and *Bambiraptor* (AMNH [American Museum of Natural History, New York City, New York, United States of America] FR 30556). The neural spine is laterally compressed and was at least as tall as the centrum. Its tip and the diapophyses and parapophyses are eroded off. A single pneumatopore is present at mid-length on each side of the centrum, as in *Deinonychus* [29] and *Bambiraptor* (AMNH FR 30556). A pneumatopore is absent in the axis of *Tsaagan* [20]. The cranial surface of the intercentrum is flat, and the caudal end of the centrum is shallowly concave. As in *Deinonychus* [29] and *Bambiraptor* (AMNH FR 30556), the centrum of the axis lacks the beveling that is characteristic of dromaeosaurid mid-cervical centra.

One cervical vertebra is completely preserved except that its neural spine is broken off (Fig. 7C). On the basis of comparison with the holotype of *Tsaagan mangas*, the first ten cervicals of which were found articulated in situ [20], we identify it as the first postaxial vertebra (cervical 3) because the diapophyses and parapophyses are not prominent. This is also true of the fourth cervical of *T. mangas*, but the shapes of the postzygapophyses and the laminae connecting them to the prezygapophyses are more similar to those of the third than the fourth cervical of *T. mangas*. The cranial surface of its centrum is not strongly beveled as are those of most other coelurosaurs, including other dromaeosaurids [29], [84]. This suggests that this part of its neck was held in less of an S-curve than in other coelurosaurs. It is amphiplatyan. Its right side is better preserved than the left and bears two short, horizontal grooves, one dorsal to the other, immediately ventral to the diapophysis. The more dorsal groove may be a pneumatopore; obscuration by matrix prevents evaluation of whether it is a foramen. The prezygapophyses are well separated and flexed. The epipophyses are distally placed and slightly overhang the postzygapophyses.

Two nearly complete posterior cervical vertebrae are present. The more cranial of the two (Fig. 7F) has been obliquely flattened so that its dorsal parts are caudally displaced. Its centrum is amphiplatyan and lacks beveling. No pneumatopores are discernible. Small parapophyses that protrude only slightly are present on the ventrolateral edge of the cranial rim of the centrum, the left more clearly visible than the right. The neural spine is broken off. The epipophyses do not reach the tips of the postzygapophyses and are located above the postzygapophyseal articular facets. The diapophyses are connected to and extend farther laterally than the prezygapophyses. A small part of the neural arch from the succeeding vertebra is preserved in articulation with the caudal end of this vertebra above the neural canal.

The other nearly complete posterior cervical vertebra is missing the neural spine, the left cranial part of the neural arch, and the left dorsolateral part of the centrum (Fig. 7G). The centrum lacks beveling and has no discernible pneumatopores. Only the right parapophysis is preserved. It is ovoid in lateral view, with its cranial end more ventral than its caudal end. The parapophysis is much larger than its counterpart on the other posterior cervical. As with the other posterior cervical, the epipophyses are above the postzygapophyseal facets and do not reach the tips of the postzygapophyses, and the diapophyses are connected to and extend farther laterally than the prezygapophyses.

Partial neural arches from two other cervical vertebrae are present. On one (Fig. 7D) the left prezygapophysis, the horizontal lamina connecting it with the left postzygapophysis, the base of the right prezygapophysis, and the base of the neural spine are preserved. The prezygapophysis is flexed. On the other (Fig. 7E) the left postzygapophysis and the base of the left prezygapophysis are preserved.

Two cervico-dorsal vertebrae are preserved, each with a hypapophysis. Neither has pneumatopores and both are amphiplatyan. In both vertebrae the parapophysis is on the anterodorsal edge rather than the anteroventral edge of the centrum, which indicates that both are from the caudal section of the series of vertebrae with hypapophyses. The vertebra with the larger hypapophysis is therefore the more cranial of these two vertebrae. The more cranial of the two (Fig. 7H) is missing the right prezygapophysis and parapophysis and all but the base of the neural spine. Its hypapophysis is about half as high as the centrum. Its parapophyses are ovoid in lateral view and slanted as in the cervical vertebra described in the preceding paragraph. They stick out farther laterally than on the cervical vertebra and are on short stalks. The one preserved prezygapophysis extends much farther laterally than the postzygapophyses and is slanted at about 45°, unlike the cervical prezygapophyses, which are not slanted. The diapophysis is about midway between the pre- and postzygapophyses. It extends much farther laterally than the zygapophyses and is about twice as long transversely as it is wide sagittally. It slants at about 30°, with its tips higher than its base.

The more posterior cervico-dorsal vertebra is missing the diapophyses, the prezygapophyses, the right postzygapophysis, and all but the bases of the parapophyses (Fig. 7I). Its hypapophysis is about one-third the height of the centrum. The postzygapophyses are much more closely spaced than in the other cervico-dorsal and the cervicals, with only a narrow notch between them. The left postzygapophysis is slanted about 45°. The neural spine is at least as tall as the centrum. Its tip is eroded away.

The neural arch of the dorsal vertebra appears to be broken off the vertebra rather than simply unfused to the centrum, because of the rough texture of its ventral surfaces. It is missing all four zygapophyses (Fig. 7J). The neural spine is broken but the height of its preserved portion is similar to that of the more posterior cervico-dorsal. The transverse processes are of similar dimensions and slant at about the same angle as those of the more posterior cervico-dorsal.

**Figure 8:**
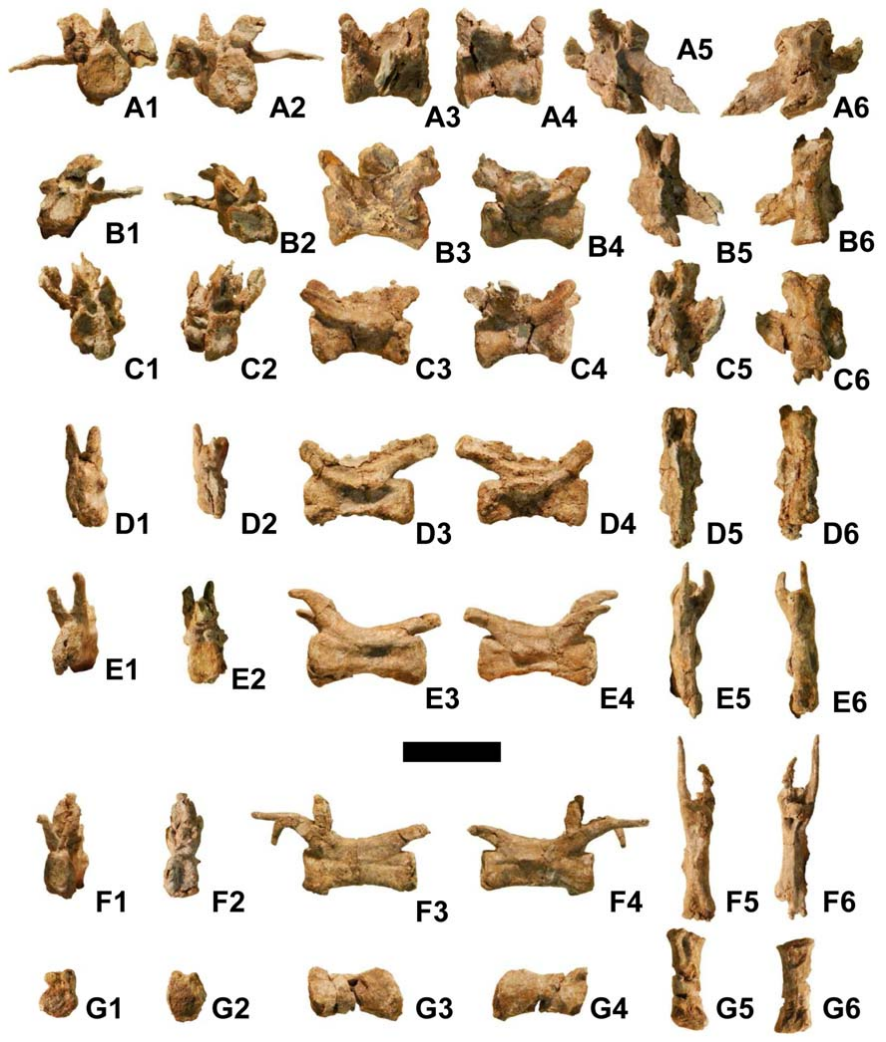
Caudal vertebrae of *Yurgovuchia doellingi* (UMNH VP 20211). (A – F)–Six consecutive caudal vertebrae from anterior and middle section of tail. (G)–Centrum of a distal caudal vertebra. Meanings of numbers on sub-figures same as in previous figure. Scale bar = 50 mm.

The cranial six of the seven preserved caudal vertebrae appear to form a consecutive series (Fig. 8). All are amphiplatyan and lack pneumatopores. In all six the postzygapophyses are more closely spaced than the prezygapophyses, and both the pre- and postzygapophyses are progressively more closely spaced in more posterior vertebrae. In the first vertebra of the series, the cranial and caudal faces of the centra are round, and the centra are round in cross-section. This resembles the condition in *Achillobator* [9]. It is unlike the condition in other dromaeosaurids, in which the proximal caudal centra are subquadrangular in cross-section, [29], [38]. The more distal caudal vertebrae of *Y. doellingi* have subquadrangular central faces.

The first (most proximal) caudal vertebra in the series (Fig. 8A) is from the transition point in the tail. In it and the next two vertebrae the transverse processes are elongate, subhorizontal, and slightly backswept, as in the proximal caudals of other dromaeosaurids [29], [85]. The transverse processes are reduced to ridges on the next three vertebrae. In the first vertebra of the series the left transverse process has broken at the base and twisted around the break in the frontal plane. The left transverse processes of the second and third vertebrae are broken off at the base.

The prezygapophyses are vertical on the first vertebra of the series and upswept in lateral view (Fig. 8A) but are not vertical on the next three vertebrae (Fig. 8B – D). The zygapophyses of the last two vertebrae in the series (Fig. 8E, F) taper quickly into narrow rods that extend far forward; their tips are broken off, but tapering suggests that they extended very little further than the broken tips. The postzygapophyses extend farther caudally than the centra in all six vertebrae. The neural spines are broken off just above the base in the first four vertebrae of the series. In the first vertebra the base of the neural spine begins about halfway down the length of the centrum (Fig. 8A). It begins progressively farther cranially on the following vertebrae and begins at the cranial margin of the neural arch in the last two vertebrae, in which the entire neural spine is preserved and is merely an extremely low ridge that extends caudally between the postzygapophyses.

On the fifth caudal vertebra in the series (Fig. 8E), a prong of bone extends vertically from between the bases of the prezygapophyses. However, it is not certain that this prong is part of the vertebra. It is likely a fragment from another bone that is currently held in place by matrix. Such a prong is absent in the caudal vertebrae of other dromaeosaurids.

One other caudal vertebra is preserved (Fig. 8G). It is significantly shorter than the posterior three caudal vertebrae of the series and is therefore probably from close to the tip of the tail. Its neural arch is missing except for the bases of the prezygapophyses. On the caudal end of its ventral surface is a sulcus. A shallower sulcus in the same location is present on the posteriormost two of the vertebrae in the caudal series. A ventral depression is also present in the distal caudal centra of *Velociraptor* [85].

In unenlagiine and microraptorian dromaeosaurids the middle and distal caudals are at least twice the lengths of those cranial to the transition point [7], [15], [25], [78]. In eudromaeosaurs the middle caudals are longer than but less than twice as long as the proximal caudals, and the distal caudals are shorter than the proximal caudals [26], [29], [85]. In these respects *Yurgovuchia* exhibits the eudromaeosaurian condition. In *Velociraptor* prezygapophyseal elongation begins with the tenth vertebra [84]. If the same is true for *Yurgovuchia*, then the series of six caudals consists of caudals 6–11.

**Figure 9:**
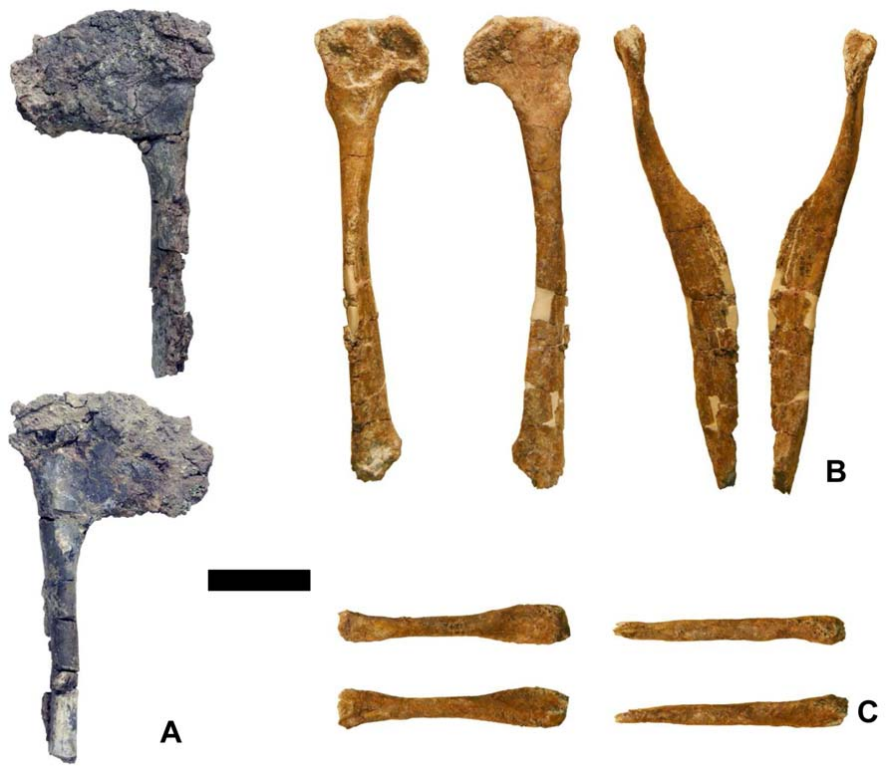
Appendicular bones of new dromaeosaurids. (A)–Left pubis of *Y. doellingi* (UMNH VP 20211) in medial (above) and lateral (below) views. (B)–Velociraptorine right pubis (UMNH VP 21752) in (left to right) medial, lateral, cranial, and caudal views. (C)–Possible velociraptorine radius(UMNH VP 21751) in four views. Scale bar = 50 mm.

The proximal end of the left pubis is preserved (Fig. 9A). The pubic shaft is perpendicular to the iliac surface of the pubis. Part of the pubic apron is preserved in the distal part of the bone. As in most dromaeosaurids [6], [7], [9], [10], [15], [29], [86], but in contrast to velociraptorines [38], a pubic tubercle is absent.

### New Pubis and Radius

These two bones specimen were found at Don’s Place, in the same layer as the holotype of *Y. doellingi*. The pubis (UMNH VP 21752) was at the opposite end of the quarry from the *Y. doellingi* bones, and the radius (UMNH VP 21751) was about halfway between the two (Fig. 3).

The shaft and proximal end of the right pubis are preserved (Fig. 9B). The preserved length of the pubis is 222 mm. The craniocaudal length of the proximal end of the pubis, perpendicular to the shaft, is 54.28 mm. The distal tip of the pubis is missing. The iliac margin of the pubis is perpendicular to the shaft. The caudal margin of the ischial peduncle is subparallel to the pubic shaft. A prominent, cranially rounded, transversely compressed tuber is present along the cranial edge of the pubis approximately 30 mm from the proximal extremity. This feature, the pubic tubercle, is a muscle and ligament attachment site [87]. It is similarly prominent and similarly shaped in the velociraptorine dromaeosaurids *Velociraptor* [38] and *Tsaagan* (cast of holotype of “*Linheraptor exquisitus*,” IVPP [Institute of Vertebrate Paleontology and Paleoanthropology, Beijing, People’s Republic of China] V 16923). A pubic tubercle as a discrete, prominent process is absent in other dromaeosaurids [6], [7], [9], [10], [15], [29], [86].

The pubic shaft is craniocaudally flattened. As in other dromaeosaurids [6], [26], [29], [85], the pubic apron is present in the distal half of the pubis. The shaft is straight, as in most other dromaeosaurids [7], [11], [25], [29], [38], [85] and unlike the pubis of some microraptorians, which is kinked backward at mid-shaft [10], [14], [15], [24]. It lacks the lateral tab at mid-shaft that is present in some microraptorians [24], [88]. The maximum transverse width of the pubic apron is 24.74 mm. The transverse width of the pubic shaft proximal to the apron and approximately halfway between the proximal end of the pubis and the proximal extremity of the apron, is 12.16 mm.

This pubis definitely comes from an individual other than the *Y. doellingi* holotype. The pubis of the latter is much larger and lacks the pubic tubercle.

The discrete pubic tubercle and the close morphological match between the pubis and those of velociraptorines [38] (cast of IVPP V 16923) indicate that the pubis is velociraptorine. If so, the dimensions of the radius are consistent with its having come from the same individual. In velociraptorines [26], [84] the radius is much shorter relative to its diameter than it is in most other dromaeosaurids [7], [9], [10], [14], [15], [29]. The radius described here is short relative to its length (Fig. 9C), as in velociraptorines. As preserved, it is 109.40 mm long and 10.31 mm wide at midshaft. Both ends are expanded (widths: 19.33 mm and 20.26 mm), but little can be said about their morphology because both tips are missing and may have been chewed off before burial.

### New Tail Skeleton

**Figure 10:**
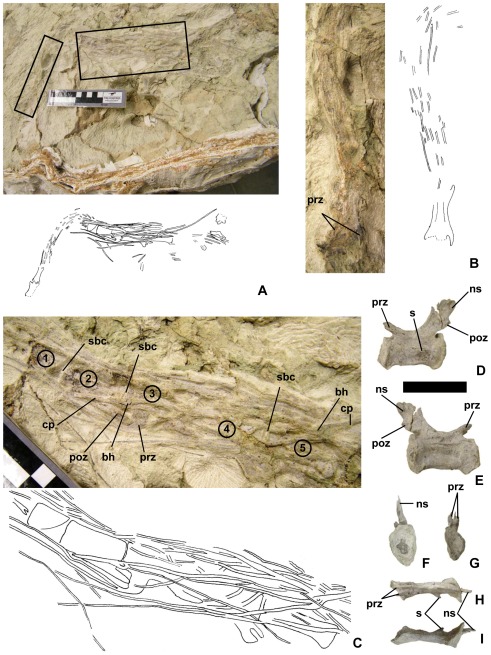
New dromaeosaurid tail skeleton (UMNH VP 20209). (A)–The tail in situ, with boxes indicating areas enlarged in B and C. (B)–Detail of A (the rectangle on the left in A). (C)–detail of A (the rectangle on the right in A). (D – I)–Isolated proximal caudal vertebra of same specimen in left lateral (D), right lateral (E), caudal (F), cranial (G), dorsal (H), and ventral (I) views. In B, anatomical anterior (cranial) is toward the bottom of the page; in C, anatomical anterior (crania) is to the left. Scale bar for D – I is 40 mm. 1–5 = “centra 1–5″ (see text), bh = body of hemal arch, ns = neural spine, poz = postzygapophysis, cp = caudal process of hemal arch, prz = prezygapophysis, sbc = space between centra.

**Figure 11:**
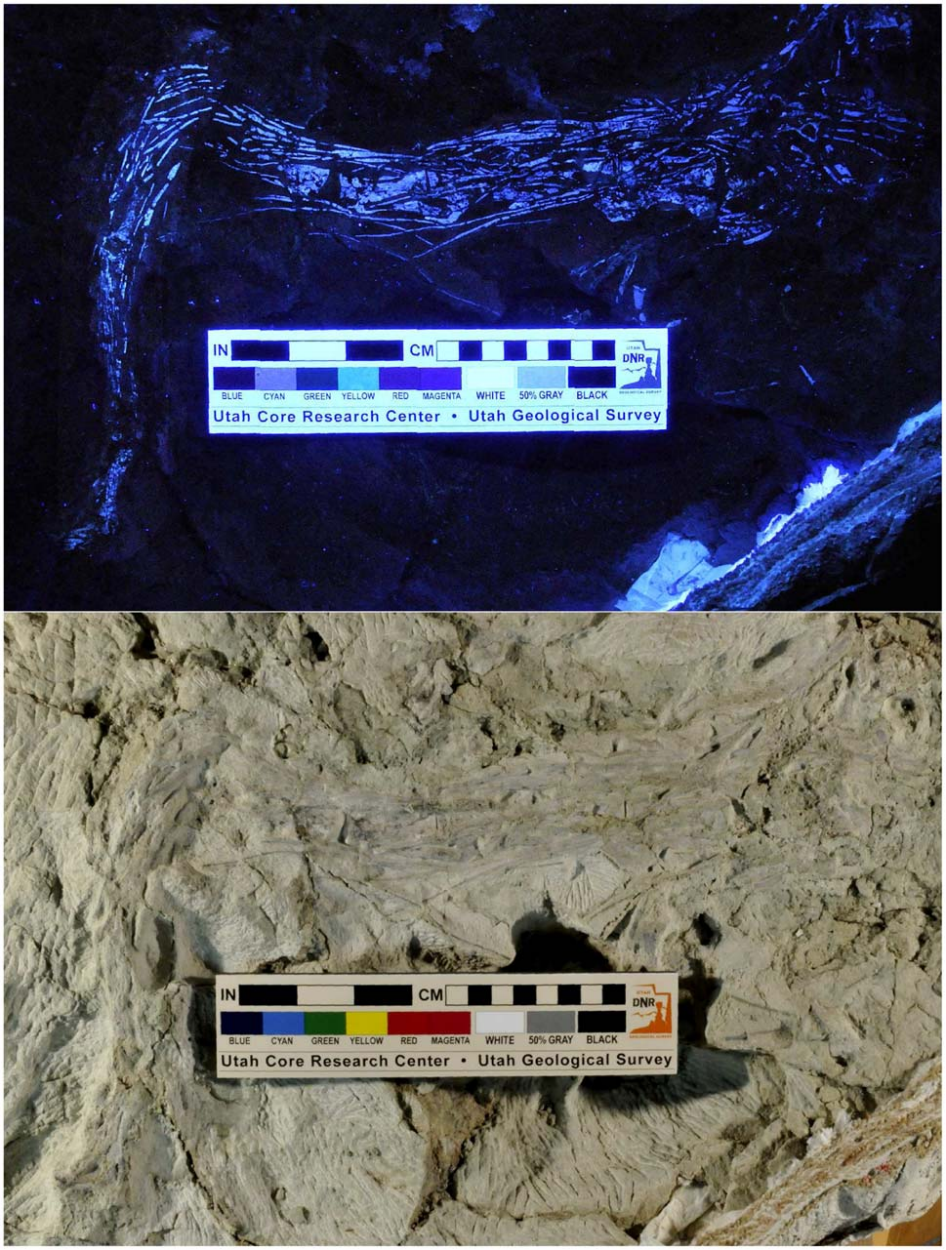
New dromaeosaurid tail skeleton (UMNH VP 20209) under ultraviolet (above) and visible (below) light.

UMNH VP 20209 consists of a proximal caudal vertebra (Fig. 10D) and a section approximately 458 mm long from a more distal section of the tail (Fig. 10A – C). The vertebrae of the latter section are bound together by a caudotheca. The bone material is nearly the same color as the matrix under natural lighting, which makes it difficult to see (Fig. 10). We have therefore included a photo of the specimen under ultraviolet light (Fig. 11) to elucidate the locations of the bones in Fig. 10. The specimen was found in a light gray sandstone layer at Andrew’s Site in Grand County, Utah (Fig. 2, 3). The site is part of the upper Yellow Cat Member (Aptian) of the Cedar Mountain Formation. The specimen’s stratigraphic position is equivalent to that of the Gaston Quarry, which is approximately 2 km to the south-southeast (Fig. 1). The Gaston Quarry yielded the holotype of the eudromaeosaur *Utahraptor ostrommaysorum*. We do not consider the new tail skeleton to represent *U. ostrommaysorum,* because the latter exhibits a hemicaudotheca, whereas the new tail skeleton exhibits a caudotheca.

The proximal caudal vertebra (Fig. 10D – I) has a centrum 45 mm long. It is amphiplatyan with a cranial surface 27 mm high×17 mm wide and a caudal surface 30 mm high×20 mm wide. Including the neural arch, the total height of the vertebra is 58 mm and its total length is 57 mm. Its prezygapophyses do not extend farther forward than the cranial surface of the centrum. The neural spine is backswept; it is present in the cranial half of the centrum only as a low ridge between the bases of the prezygapophyses. The postzygapophyses emanate as small facets from the caudal edge of the neural spine at the same height as the tips of the prezygapophyses. On the left side of the centrum is a pathological bony spur. No trace of a transverse process is present on either side. Immediately dorsal to the tiny ridge on the left is a longitudinal sulcus. Such a sulcus is also present at the same height on the right side.

The distal section of tail is sharply bent at approximately a right angle, 218 mm from its cranial end (Fig. 10A). The tail is twisted at the bend so that cranial to the bend the tail is preserved in dorsal view, and caudal to it the tail is preserved in right lateral view. The caudotheca has unraveled somewhat, and some hemal arches are displaced. Cranial to the bend at least five centra are present, judging from the lengths of the measurable centra caudal to the bend. The caudotheca is absent cranial to the third vertebra in this series and surrounds that vertebra and all vertebrae caudal to it. Enough is visible of the prezygapophyses of the second vertebra in this series to ascertain that they do not extend farther forward than the centrum (Fig. 10B).

Caudal to the bend the caudal end of a centrum (hereafter called centrum 1) and four more consecutive centra (hereafter called centra 2–5) are visible. Only centrum 2 can be measured with certainty. It is 41 mm long and is cranially 16 mm tall. Centra 3–5 are estimated to be 40, 43, and 46 mm long respectively. All are of similar height.

In a few cases elongated prezygapophyses can be traced to their respective vertebrae. Their lengths cannot be measured with certainty because their tips may be broken off. The longest visible section of a prezygapophysis is from centrum 5. It is bifurcated at its base, and the two tines remain close to each other, nearly appressed, through half their preserved lengths. The longer (as preserved) of the two tines extends 122 mm beyond the centrum (approximately 2.7 times the length of the centrum). One tine of a prezygapophysis is traceable to centrum 3. As preserved, it extends 106 mm beyond the centrum. In contrast, the postzygapophysis of centrum 3 extends only approximately 17 mm beyond the centrum. That of centrum 4 extends only approximately 11 mm beyond the centrum.

A detached hemal arch lies on the right side of the tail, with its body on the anterodorsal corner of centrum 3 (Fig. 10C). It has been turned around so that its caudal process extends cranially. That process extends 30 mm beyond the body and is not bifurcated at the tip. There are two cranial projections, neither of which is itself bifurcated, the bases of which are joined at the body of the hemal arch. The longer (as preserved) of these two projections extends 82 mm from the body of the bone.

In the new specimen, all the proximal caudals with transverse processes are missing. Using a conservative estimate of four missing caudals with transverse processes, we see that the first caudal vertebra contributing to the caudotheca is no farther cranial than the eighth caudal vertebra and the caudotheca extends no farther forward than the seventh.

### Phylogenetic Analysis

The phylogenetic analysis recovered 1217 most-parsimonious trees with 1304 steps. For these trees, the consistency index is 0.3758, the homoplasy index is 0.6242, the retention index is 0.8124, and the rescaled consistency index is 0.3053.

**Figure 12:**
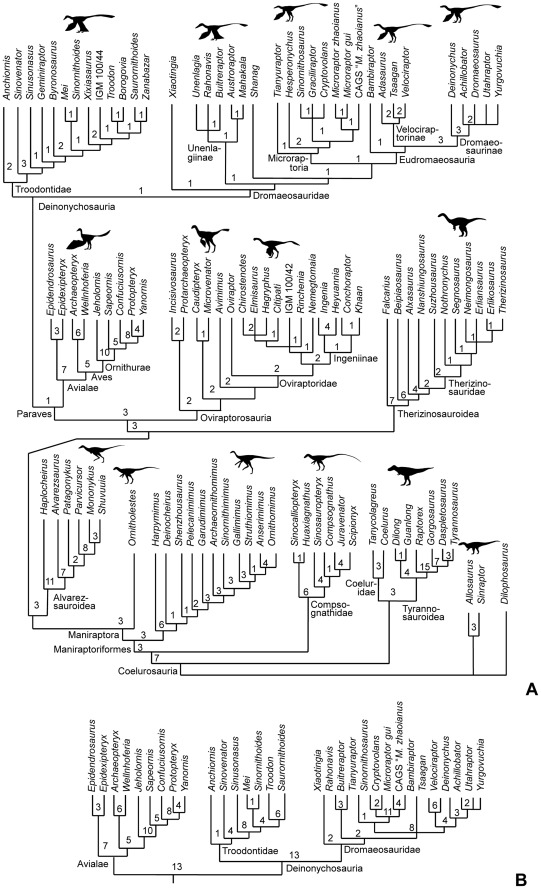
Phylogeny of Coelurosauria, as found by this study. Numbers at base of each clade are decay indices (Bremer support). (A)–Phylogeny recovered when fragmentary deinonychosaurian OTUs are included. (B)–Paravian portion of the phylogeny recovered when several fragmentary deinonychosaurian OTUs are deleted.

The phylogeny of Coelurosauria recovered here (Fig. 12A) matches that found using the previous version of the phylogenetic data matrix [36]. Within Paraves, it differs from some recent phylogenetic analyses in the following ways. Here, *Xiaotingia* is placed at the base of Dromaeosauridae, whereas a previous analysis placed it with *Anchiornis* and *Archaeopteryx* in a clade that formed the sister taxon to Deinonychosauria [89]. Here, *Shanag* is placed at the base of the sister clade of Unenlagiinae, whereas previous analyses placed it at the base of Unenlagiinae itself [21], [22]. Here, *Mahakala* is placed within Unenlagiinae, whereas a previous analysis placed it at the base of Dromaeosauridae [21].

*Yurgovuchia* is found to be part of a clade within Dromaeosaurinae that includes *Utahraptor*, *Achillobator*, and *Dromaeosaurus* but excludes *Deinonychus*. Relationships within this clade are uncertain. Synapomorphies of Deinonychosauria and its sub-clades that are confirmed to be present on *Yurgovuchia* are given in Table 3.

**Table 3:** Synapomorphies of Deinonychosauria and its sub-clades that are confirmed to be present in *Yurgovuchia doellingi*.

Clade	Character	State number and description
Deinonychosauria	160	0: Cervical epipophyses above zygapophyseal facets
Dromaeosauridae	174	1: Posterior trunk parapophyses distinctly projected on pedicels (ACCTRAN only)
Unenlagiinae + (*Shanag* + (Microraptoria + (Eudromaeosauria)))	174	1 (DELTRAN only)
Microraptoria + Eudromaeosauria	180	0: Anterior dorsal transverse processes long and thin
	165	0: Cervical carotid processes absent (ACCTRAN only)
Velociraptorinae + Dromaeosauridae	165	0: (DELTRAN only)
	193	2: Mid-caudal centra between 1.3× and 2× as long as proximal centra
*Yurgovuchia* + *Achillobator* + *Utahraptor* + *Dromaeosaurus*	162	0: Anterior cervical centra subcircular or square in cranial view
	195	3: Prezygapophyses of distal caudal vertebrae extended in length but not longer than several centra

Character and state numbers are as given in Appendix S1. Unless otherwise mentioned, in each case the state is a synapomorphy of the clade under both accelerated and delayed transformation. ACCTRAN = accelerated transformation. DELTRAN = delayed transformation.

When deinonychosaurian OTUs known only from fragmentary material are deleted from the analysis, the analysis yields 54 most-parsimonious trees of 1265 steps. For these trees, the consistency index is 0.3866, the homoplasy index is 0.6134, the retention index is 0.8083, and the rescaled consistency index is 0.3125. Decay indices for several deinonychosaurian clades are increased, in some cases dramatically, in comparison to the analysis that included fragmentary taxa (Fig. 12B). The topology of the strict consensus tree is nearly identical to that produced without deletion of fragmentary taxa. The only change is that *Bambiraptor* is no longer at the base of Eudromaeosauria in the consensus tree but is part of an unresolved trichotomy with Eudromaeosauria and Microraptoria.

### Tail Evolution Scenario

**Figure 13:**
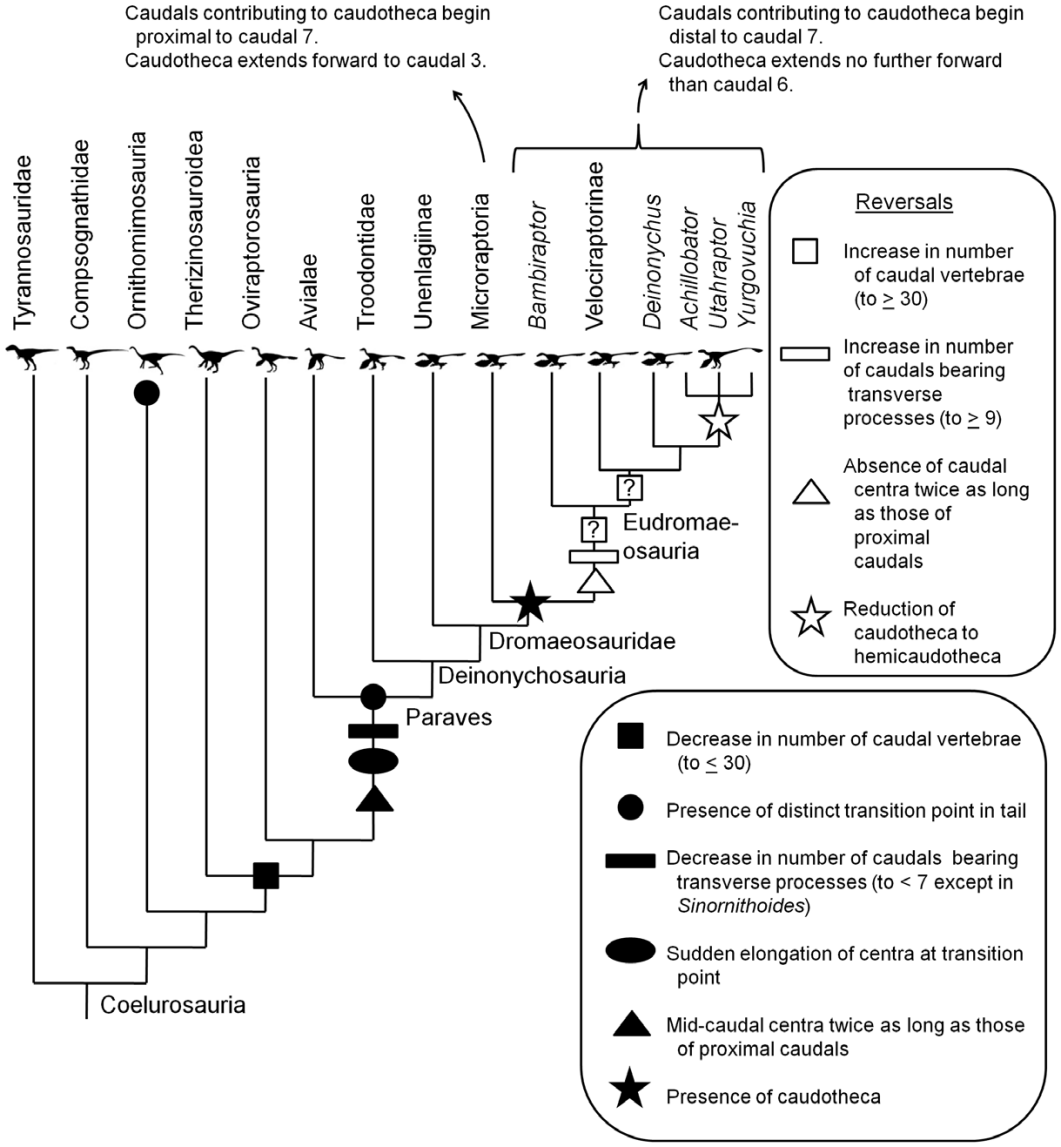
Changes in character states of the tail, mapped onto coelurosaurian theropod phylogeny.

Comparison of data from a phylogenetically broad spectrum of coelurosaurian theropod taxa (Table 1) reveals several evolutionary changes in the coelurosaurian tail (Fig. 13). Several of these changes include reversals within Dromaeosauridae. The changes are: (1) a decrease in the number of caudal vertebrae early in coelurosaurian phylogeny, followed by an increase in Eudromaeosauria, (2) a decrease in the number of caudals bearing transverse processes in Paraves, followed by an increase in this number in Eudromaeosauria, (3) elongation of middle caudal vertebrae to over twice the length of the first few caudals in Paraves, followed by a reversal in Eudromaeosauria, (4) acquisition of a caudotheca in Microraptoria + Eudromaeosauria, followed by its replacement by a hemicaudotheca in Dromaeosaurinae, (5) a difference between Microraptoria and Eudromaeosauria in the location of the first caudal vertebra with processes that contribute to the caudotheca, and (6) a difference between Microraptoria and Eudromaeosauria in the location of the cranialmost extent of the caudotheca.

In Microraptoria transverse processes are present only up to Cd (caudal vertebra) 6, the first caudal vertebra contributing to the caudotheca is no farther cranial than Cd 6, and the caudotheca extends forward to Cd 3 or 4. In Eudromaeosauria transverse processes are present up to Cd 10, the first caudal vertebra contributing to the caudotheca is no further cranial than Cd 10, and the caudotheca extends no farther forward than Cd 6. The new specimen described here that is represented only by a tail skeleton exhibits the eudromaeosaurian condition.

## Discussion

The three new dromaeosaurid specimens described here add to the known dinosaurian diversity of the upper and lower Yellow Cat Member of the Cedar Mountain Formation. The therizinosauroid *Falcarius utahensis* [3] and the small, predatory theropod *Geminiraptor suarezarum* [4] have previously been described from the lower Yellow Cat. To that theropod paleofauna we now add *Yurgovuchia doellingi* and the velociraptorine dromaeosaurid represented by the new pubis. From the upper Yellow Cat the large dromaeosaurid *Utahraptor ostrommaysorum* has previously been described [5], as has the small theropod *Nedcolbertia justinhofmanni* [2]. To that theropod paleofauna we now add the new, unnamed eudromaeosaur represented by the new tail skeleton UMNH VP 20209.

According to our phylogenetic results, *Y. doellingi* and *U. ostrommaysorum* are both members of the subfamily Dromaeosaurinae. The new pubis represents the subfamily Velociraptorinae. During the Late Cretaceous Asia was also simultaneously inhabited by dromaeosaurines (*Achillobator*) [9] and velociraptorines (*Tsaagan*, *Velociraptor*) [20], [28]. In addition, microraptorian dromaeosaurids are known from the Lower Cretaceous of Asia [10], [12], [14], [15], [18], [25], [88] and the Upper Cretaceous of North America [24]. This shows that transcontinental dispersal events occurred during the Cretaceous Period for all three dromaeosaurid clades.

Another important aspect of the phylogeny recovered here is the phylogenetic separation between *Archaeopteryx* (at the base of Aves), *Xiaotingia* (at the base of Dromaeosauridae), and *Anchiornis* (at the base of Troodontidae). The analysis that placed these three genera together in a clade of their own [89] used a modified version [48] of a phylogenetic data matrix that was published in 2007 [90]. That matrix lacked two sets of updates that were added later; one set of updates was included in a 2010 version of the matrix [91] and a further set of updates was included in a 2011 version of the matrix [36], in both cases due to recent examination of specimens by PS. Among the taxa with significant amounts of changed data due to post-2007 examination of specimens are the birds *Jeholornis*, *Sapeornis*, *Confuciusornis*, and *Yanornis*; the troodontids *Anchiornis*, *Sinusonasus*, *Mei*, and *Sinornithoides*; and the dromaeosaurids *Buitreraptor*, *Sinornithosaurus*, *Microraptor zhaoianus*, *M. gui*, *Bambiraptor*, *Tsaagan*, *Velociraptor*, *Deinonychus*, and *Utahraptor*. We therefore consider the matrix used here particularly reliable for placement of paravian taxa and therefore doubt the placement of *Archaeopteryx*, *Xiaotingia*, and *Anchiornis* in a clade of their own. The results of the phylogenetic analysis with fragmentary OTUs deleted yields a high decay index for Avialae (with *Archaeopteryx* therein), which indicates strong support for a close relationship between *Archaeopteryx* and other birds (Fig. 12B). It also yields a high decay index for Deinonychosauria (with *Anchiornis* and *Xiaotingia* therein), which indicates strong support for phylogenetic separation of *Archaeopteryx* from *Anchiornis* and *Xiaotingia* (Fig. 12B).

Coding of character states of the new pubis inspired a reevaluation of the orientation of dromaeosaurid pubes. Previous authors have considered the pubis to be retroverted in *Velociraptor* and other eudromaeosaurs [38]. However, in Paraves the pubic surface of the pubic peduncle of the ilium is horizontal [7], [9], [29], [38], [49], [50]. This means that if the iliac surface of the pubis is perpendicular to the pubic shaft, then the pubic shaft was vertical. PS has confirmed this in the dromaeosaurids *Rahonavis* and *Unenlagia* by manual articulation of originally disarticulated pelves. The iliac surface of the pubis is perpendicular to the pubic shaft in the eudromaeosaurs *Velociraptor* [85] and *Achillobator* [9] and the basal microraptorian *Tianyuraptor* [25]. Their pubes were therefore vertical, not retroverted. The retroversion of their pubes in articulated specimens [25], [38], [85] is due to disarticulation of the pubes from the ilia and subsequent rotation of the pubes. A similar situation is present in *Archaeopteryx*. Its pubes have become disarticulated from the ilia and rotated into a retroverted position in most specimens [49], [92], [93]; the pubes are vertical in the one specimen of *Archaeopteryx* in which they retain articulation with the ilia [94]. The morphology of the pubis of *Yurgovuchia* and the new velociraptorine specimen described here indicates that they, too, were vertical. Advanced microraptorians are the only dromaeosaurids in which the pubes are retroverted. In advanced microraptorians the pubic shafts are at a strong angle to the iliac surface of the pubis [10], [15], and an articulated specimen demonstrates that this causes their pubes to be retroverted [15].

The morphological dichotomy between the tails of microraptorians and eudromaeosaurs relate to a difference in the location of the transition point between the two clades. The transition point occurs farther caudally in the tail in Eudromaeosauria than it does in Microraptoria, so that the proximal segment of the tail contains a larger number of vertebrae in Eudromaeosauria. This allows the proximal segment of the tail to swing through a greater arc in Eudromaeosauria if the zygapophyseal orientations of the proximal tail segment are uniform between the two clades. Typically, eudromaeosaurs are larger and have more robust skeletons than microraptorians, and it is plausible that the smaller range of motion of the microraptorian tail was insufficient to accommodate the greater body mass of the eudromaeosaurs during sudden changes of direction during fast locomotion or other vigorous activity. That is, the small microraptorians could accommodate having a very stiff tail, but at larger body sizes dromaeosaurids needed greater tail flexibility to maintain balance.

Although a hemicaudotheca looks like an intermediate condition in the evolution of a caudotheca, the results of the phylogenetic analysis show that this is not so. The clade exhibiting a hemicaudotheca is nested within the caudothecate group and has four successive caudothecate sister groups: *Deinonychus*, Velociraptorinae, *Bambiraptor*, and Microroaptoria (Fig. 12). This indicates that the caudotheca appeared first and then became reduced into a hemicaudotheca in advanced dromaeosaurines (Fig. 13).

It is plausible that a hemicaudotheca allowed more tail flexibility than a caudotheca because of reduction in the lengths of the bony processes enclosing the caudal centra. If, as postulated above, larger dromaeosaurids required greater tail flexibility to maintain balance during vigorous activity, then a more-flexible hemicaudotheca would be more advantageous than a less-flexible caudotheca to the largest dromaeosaurids. As it happens, the largest dromaeosaurids, *Utahraptor* and *Achillobator*, exhibit a hemicaudotheca. This supports the hypothesis that the reduction of the caudotheca into a hemicaudotheca was a prerequisite for the evolution of large size in Eudromaeosauria.

## Supporting Information

Appendix S1 **Character List for Phylogenetic Analysis of Coelurosauria.**
(DOC)

Appendix S2 **Phylogenetic Data Matrix for Phylogenetic Analysis of Coelurosauria.**
(DOC)
