# Correction: New Dromaeosaurids (Dinosauria: Theropoda) from the Lower Cretaceous of Utah, and the Evolution of the Dromaeosaurid Tail

**DOI:** 10.1371/annotation/acddcd7d-0e2e-4abb-acbf-d5552fa286f8

**Published:** 2012-09-06

**Authors:** Phil Senter, James I. Kirkland, Donald D. DeBlieux, Scott Madsen, Natalie Toth

The image for Figure 5 is incorrect. The correct image for Figure 5 can be seen here:

**Figure:**
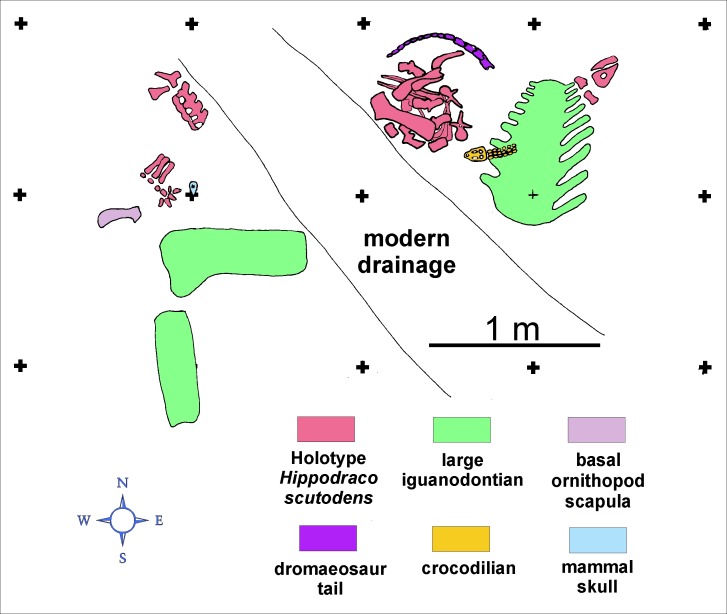


[^] 

The current legend for Figure 5 is correct.

