# Supplementary material for: New Dromaeosaurids (Dinosauria: Theropoda) from the Lower Cretaceous of Utah, and the Evolution of the Dromaeosaurid Tail
Source: PLoS One. 2012 May 15;7(5):e36790. doi: 10.1371/journal.pone.0036790 (PMC3352940; doi:10.1371/journal.pone.0036790)
Supplement: Appendix S1 — Character List for Phylogenetic Analysis of Coelurosauria. (DOC) [file pone.0036790.s001.doc]

**Appendix S1: Character List for Phylogenetic Analysis of Coelurosauria**

Here, coelurosaurian forelimb digits are identified as digits 1, 2, and 3, not as digits 2, 3, and 4. Identification of the digits as 1, 2, and 3 is congruent both with paleontological data and with the results of studies on avian embryology and the genes governing avian digit identity [1,2].

Italics indicate new characters, character states, or changes of wording with respect to the previous character list [3].

**Character List**

1. Skull 90 – 115% femoral length (0) or less than 60% (1) or between 60 – 85% (2) or > 130% (3) [3].

2. Length of preorbital region of cranium > height at anterior edge of preorbital bar (exclusive of midline sagittal ridge, if any) (0) or < height at anterior edge of preorbital bar (1) [4].

3. Length of preorbital region of cranium < 3x height of orbit (0) or > 3x height of orbit [3].

4. Height of skull (minus mandible) at middle of naris more than half the height of skull at middle of orbit (0) or less than half (1) [4].

5. Snout (without mandible) is not broadly rounded and semicircular in cross-section (0) or is, and is transversely broad (1) [3].

6. Premaxillary symphysis acute, V-shaped (0) or rounded, U-shaped (premaxillary tooth row, if any, nearly transverse) (1) (reworded from reference 5).

7. Body of premaxilla ventral to external naris (0) or rostral to external naris (1). [3] (illustrated in reference 3, fig. S1a)

8. Premaxilla does not (0) or does taper rostrally in lateral view, coming to a sharp rostral point (1) (Reworded from reference 4).

9. Anteroventral corner of premaxilla does not (0) or does form an acute, ventrally oriented point in lateral view (1) (reworded from reference 6).

10. Crenulate margin on buccal edge of premaxilla absent (0) or present (1) [5].

11. External naris an elongate oblong, with long axis diagonal in lateral view (0) or an elongate oblong with long axis subhorizontal (1) or a short oblong, with length not much greater than height, with long axis subhorizontal (2) [3]. This character replaces a previous one: “Internarial bar rounded (0) or flat (1) [5].” The internarial bar appears flat when the long axis of the external naris is subhorizontal. (illustrated in reference 3, fig. S1b)

12. Dorsal margin of naris below level of dorsal margin of orbit (0) or above (1) [4].

13. Caudal margin of naris farther rostral than (0), or nearly reaching or overlapping (1), the rostral border of the antorbital fossa [7].

14. Length of external naris similar to that of orbit (0) or much shorter than orbit (1) [8].

15. Maxillary process of premaxilla contacts nasal to form posterior border of nares (0) or maxillary process reduced so that maxilla participates broadly in external naris (1) or maxillary process of premaxilla extends posteriorly to separate maxilla from nasal posterior to nares (2) [5].

16. Antorbital fossa anteriorly bounded by maxilla (0) or by premaxilla (1) [6].

17. Length of maxilla taken up by internal antorbital fenestra: > 45% of total maxilla length (0) or between 33% and 45% of total maxilla length (1) or < 33% of total maxilla length (2) [3].

18. Shape of rostral end of maxilla, anterior to antorbital fossa (sometimes discernible by shape of premaxilla when maxilla is unknown): with anterior border that meets the ventral border at a slant and without sinusoidal border beneath external naris, (0), or with tall, vertical anterior border (1), or with sinusoidal anterior border beneath external naris such that below naris is an extension with a vertical anterior border (2) [3]. (illustrated in reference 3, fig. S1c)

19. Extension of maxilla beneath external naris (nonexistent except in *state 2* of previous character): dorsoventrally narrow (0) or dorsoventrally tall (1) [3]. (illustrated in reference 3, fig. S1d; italicized phrase indicates correction of typographical error in reference 3).

20. Maxillary antorbital fossa: small, from 10% to less than 40% of the rostrocaudal length of the antorbital cavity (0), large, greater than 40% of the rostrocaudal length of the antorbital cavity (1) [9].

21. Antorbital fossa without distinct rim ventrally and anteriorly (0) or with distinct rim composed of a thin wall of bone (1) [5].

22. Antorbital fossa is not (0) or is peppered with small pits (1) [3].

23 .Anterior border of antorbital fossa broadly rounded (0) or steep and straight, meeting the horizontal ventral edge at a sharply defined anteroventral corner (1) or narrow and rounded (2) [3]. (illustrated in reference 3, fig. S1e)

24. Ventral to internal antorbital fenestra, antorbital fossa is dorsoventrally deep (0) or dorsoventrally shallow (1) [3]. (illustrated in reference 3, fig. S1f)

25. Ventral to antorbital fossa, lateral surface of maxilla is dorsoventrally shallow (0) or dorsoventrally deep (1) [3]. (illustrated in reference 3, fig. S1g)

26. Promaxillary fenestra absent (0) or present (1) [5].

27. Maxillary fenestra absent (0) or present (1) [5].

28. Maxillary fenestra large and round or ovoid and not dorsally displaced (0), a large, craniocaudally elongate oblong, > 3x longer than high, not dorsally displaced (1), a small, craniocaudally elongate slit, not dorsally displaced (2), or a dorsally displaced opening (3) (reworded from reference 10).

29. Maxillary fenestra situated at rostral border of antorbital fossa (0) or situated posterior to rostral border of fossa (1) [5].

30. Area of antorbital fenestra greater than that of orbit (0) or less than that of orbit (1) [10].

31. Secondary palate short (0) or long, with extensive palatal shelves on maxilla (1) [4].

32. Palatal shelf of maxilla flat (0) or with midline ventral ‘tooth-like’ projection (1) [5].

33. Narial region apneumatic or poorly pneumatized (0) or with extensive pneumatic fossae, especially along posterodorsal rim of fossa (1) [5].

34. Nasals at least as long as frontals (0) or shorter than frontals (1) [4].

35. Nasal surface: smooth (0), rugose (1) [9].

36. Nasal fusion: absent, nasals separate (0), present, nasals fused together (1) [9].

37. Midsagittal ridge formed by dorsal displacement of midline of frontals, nasals, and premaxillae absent (0) or present (1) [4].

38. Orbit round in lateral or dorsolateral view (0) or dorsoventrally elongate (1) [5].

39. Supraorbital crest on lacrimal in adult individuals absent (0) or dorsal crest above orbit (1) [5].

40. Lateral expansion on lacrimal, anterodorsal to orbit: absent (0) or present (1) [5].

41. Enlarged foramen or foramina opening laterally at the angle of the lacrimal, absent (0) or present (1) [5].

42. Lacrimal posterodorsal process absent (0) or horizontal and similar in length to the jugal process (1) or horizontal and much shorter than the jugal process or (2) present and vertical (3) (reworded from reference 4).

43. Prefrontal large, dorsal exposure similar to that of lacrimal (0) or greatly reduced in exposure (1) or without exposure (2) (modified from reference 5).

44. Postorbital bar present (0) or absent (1) [3].

45. Postorbital bar: narrow, similar in breadth to preorbital bar (0) or much broader than preorbital bar (1) [3].

46. Anterior process of postorbital projects into orbit (0) or does not project into orbit (1) [5].

47. Postorbital in lateral view with subhorizontal anterior (frontal) process (0) or frontal process diagonal (anterior tip of process higher than base of process) (1) [4].

48. Postorbital bar parallels quadrate, lower temporal fenestra rectangular in shape (0) or jugal and postorbital approach or contact quadratojugal to constrict lower temporal fenestra (1) [5].

49. Jugal pneumatic recess in posteroventral corner of antorbital fossa present (0) or absent (1) [5].

50. Suborbital process of jugal dorsoventrally stout, *and not as in state 2* (0) or elongate and dorsoventrally narrow (1) or dorsoventrally stout, with caudal end dorsoventrally taller than cranial end, so that orbital margin slopes downward cranially (2) (modified from reference 10).

51. Extreme dorsoventral thickening of suborbital process of jugal (so that the process is high relative to its length and much dorsoventrally thicker than the lower temporal bar): absent (0) or present (1) [3].

52. Contribution of jugal to orbital margin: short (0) or extensive, ventral ends of postorbital and lacrimal widely separated (1) [3]

53. Suborbital process of jugal transversely flattened, platelike (0) or rodlike (1) [11].

54. Sublacrimal process of jugal dorsoventrally expanded (taller than suborbital bar of jugal) (0) or not dorsoventrally expanded (1) [11].

55. Ventral border of jugal + maxilla in lateral view: approximately straight (0) or strongly arched, concave ventrally (1) [3].

56. Jugal quadratojugal process tall beneath lower temporal fenestra, craniocaudally short (0) or dorsoventrally narrow and craniocaudally elongate (1) (reworded from reference 5).

57. Quadratojugal without horizontal process posterior to ascending process (reversed “L” shape) (0) or with process (i.e., inverted ‘T’ or ‘Y’ shape) (1) [5].

58. Descending process of squamosal parallels quadrate shaft (0) or nearly perpendicular to quadrate shaft (1) [5].

59. Supratemporal fenestra bounded laterally and posteriorly by the squamosal (0) or supratemporal fenestra extended as a fossa on to the dorsal surface of the squamosal (1) [12].

60. Posterolateral shelf on squamosal overhanging quadrate head absent (0) or present (1) [5].

61. Quadrate head covered by squamosal in lateral view (0) or quadrate cotyle of squamosal open laterally exposing quadrate head (1) [5].

62. Quadrate vertical (0) or strongly inclined anteroventrally so that mandibular end lies far forward of dorsal end (1) [5].

63. Quadrate solid (0) or hollow, with depression on posterior surface (1) [5].

64. Lateral border of quadrate shaft straight (0) or with lateral tab that touches squamosal and quadratojugal above an enlarged quadrate foramen (1) [5].

65. Length of ventral border of infratemporal fenestra comparable to that of orbit (0) or much shorter (1) [4].

66. Forebrain small and narrow (0) or large and triangular (strongly domed frontals are evidence for state 1 if an endocast is not available, although a lack of frontal doming does not necessarily indicate state 0) (1) [11].

67. Contribution of frontal to orbital margin: absent or tiny (0) or substantial (1) [3].

68. Frontals narrow anteriorly as a wedge between nasals (0) or end abruptly anteriorly, suture with nasal transversely oriented (1) or suture with nasals W-shaped (2) [4].

69. Frontal edge smooth in region of lacrimal suture (0) or edge notched (1) [13].

70. Frontal postorbital process (dorsal view): smooth transition from orbital margin (0) or with lateral prong that is sharply demarcated from orbital margin (1) (reworded from reference 13).

71. Anterior emargination of supratemporal fossa on frontal straight or slightly curved (0) or strongly sinusoidal and reaching onto postorbital process (1) [13].

72. Parietals shorter than frontals (0) or longer (1) [10].

73. Dorsal surface of parietals flat, lateral ridge borders supratemporal fenestra (0) or parietals dorsally convex with very low sagittal crest along midline (1) or dorsally convex with well developed sagittal crest (2) [5].

74. Parietals separate (0) or fused (1) [5].

75. Depression (possibly pneumatic) on ventral surface of postorbital process of laterosphenoid absent (0) or present (1) [12].

76. Otosphenoidal crest vertical on basisphenoid and prootic, and does not border an enlarged pneumatic recess (0) or well developed, crescent shaped, thin crest forms anterior edge of enlarged pneumatic recess (1) [5]. This structure forms the anterior, and most distinct, border of the “lateral depression” of the middle ear region [14,15] of troodontids and some extant avians.

77. Crista interfenestralis confluent with lateral surface of prootic and opisthotic (0) or distinctly depressed within middle ear opening (1) [5].

78. Depression for pneumatic recess on prootic absent (0) or present as dorsally open fossa on prootic/opisthotic (1) or present as deep, posterolaterally directed concavity (2) [5]. The dorsal tympanic recess referred to here is the depression anterodorsal to the middle ear on the opisthotic, not the recess dorsal to the crista interfenestralis within the middle ear as seen in *Archaeopteryx lithographica*, *Shuvuuia deserti* and Aves.

79. Accessory tympanic recess dorsal to crista interfenestralis absent (0) small pocket present (1) or extensive with indirect pneumatization (2) [5].

80. Subotic recess (pneumatic fossa ventral to fenestra ovalis) absent (0) or present (1) [5].

81. Basisphenoid recess present between basisphenoid and basioccipital (0) or entirely within basisphenoid (1) or absent (2) [5].

82. Posterior opening of basisphenoid recess single (0) or divided into two small, circular foramina by a thin bar of bone (1) [5].

83. Base of cultriform process (parasphenoid rostrum) not highly pneumatized (0) or base of cultriform process expanded and pneumatic (parasphenoid bulla) (1) [5].

84. Basal tubera set far apart, level with or beyond lateral edge of occipital condyle and/or foramen magnum (may connected by a web of bone or separated by a large notch) (0) or tubera small, directly below condyle and foramen magnum, and separated by a narrow notch (1) [5].

85. Basipterygoid processes ventral or anteroventrally projecting (0) or lateroventrally projecting (1) [5].

86. Basipterygoid processes well developed, extending as a distinct process from the base of the basisphenoid (0) or processes abbreviated or absent (1) [5].

87. Basipterygoid processes solid (0) or processes hollow (1) [5].

88. Basipterygoid recesses on dorsolateral surfaces of basipterygoid processes absent (0) or present (1) [5].

89. Exits of C. N. X-XII flush with surface of exoccipital (0) or cranial nerve exits located together in a bowl-like basisphenoid depression (1) [5].

90. Paroccipital process elongate and slender, with dorsal and ventral edges nearly parallel (0) or process short, deep with convex distal end (1) [5].

91. Paroccipital process straight, projects laterally or posterolaterally (0) or distal end curves ventrally, pendant (1) [5].

92. Paroccipital process with straight dorsal edge (0) or with dorsal edge twisted rostrolaterally at distal end (1) [13].

93. Caudal (posterior) tympanic recess absent (0) present as opening on anterior surface of paroccipital process (1) or extends into opisthotic posterodorsal to fenestra ovalis, confluent with this fenestra (2) [5].

94. Foramen magnum smaller than or subequal to size of occipital condyle (0) or larger than occipital condyle (1) [6] [4].

95. Foramen magnum subcircular, slightly wider than tall (0) or oval, taller than wide (1) [16].

96. Occipital condyle without constricted neck (0) or subspherical with constricted neck (1) [5].

97. Basioccipital without pneumatization on occipital surface (0) or with subcondylar recess (1) [5].

98. Flange of pterygoid well developed (0) or reduced in size or absent (1) [5].

99. Palatine and ectopterygoid separated by pterygoid (0) or contact (1) [13].

100. Ectopterygoid lateral to pterygoid (0) or rostral to pterygoid (1) [17].

101. Palatine-pterygoid-ectopterygoid bar does not (0) or does (1) arch below ventral cheek margin [17].

102. Ectopterygoid with constricted opening into fossa (0) or with open ventral fossa in the main body of the element (1) [5].

103. Dorsal recess on ectopterygoid absent (0) or present (1) [5].

104. Palatine tetraradiate, with jugal process (0) or palatine triradiate, jugal process absent (1) [5].

105. Suborbital fenestra similar in length to orbit (0) or about half or less than half orbital length (1) or absent (2) [4].

106. Strong bend in ventral margin of mandible such that with ventral margin of dentary horizontal, ventral margin of angular is strongly slanted (with posterior end higher than anterior end): absent (0) or present (1) [3]. (illustrated in reference 3, fig. S1h)

107. Symphyseal region of dentary broad and straight, paralleling lateral margin (0) or medially recurved slightly (1) or strongly recurved medially (2) [5].

108. Dentary symphyseal region in line with main part of buccal edge (0) or abruptly downturned at rostral end (1) or dentary ramus gradually, weakly downturned through its length (2) [4].

109. Kink and downward deflection in dentary buccal margin at rostral end of dentary: absent (0) or present (1) (reworded from reference 18).

110. Dentary ramus elongate (0) or shortened, not much longer than tall (1) [4].

111. Dentary not bowed (0) or bowed (concave dorsally) (1) [10].

112. Posterior end of dentary without posterodorsal process dorsal to mandibular fenestra (0) or with dorsal process above anterior end of mandibular fenestra (1) or with elongate, strongly arched dorsal process extending over most of fenestra (2) [5].

113. Dentary without posteroventral process extending to posterior end of external mandibular fenestra (0) or with such a process (1) [6].

114. Labial face of dentary flat (0) or with lateral ridge and inset tooth row (1) [5].

115. Nutrient foramina on external surface of dentary superficial (0) or lie within deep groove (1) [5].

116. Ventral surface of dentary straight or nearly straight (0) or descends strongly posteriorly (1). [12]

117. Dorsoventral depth of surangular is (0) or is not greater than that of dentary ramus (1) [8].

118. Surangular without coronoid prominence (0) or with coronoid prominence (1) (reworded from reference 5).

119. Horizontal shelf on the lateral surface of the surangular, rostral and ventral to the mandibular condyle: absent or faint ridge (0), prominent and extending laterally (1) [9].

120. *Large* foramen in lateral surface of surangular rostral to mandibular articulation, absent (0) or present (1) [5].

121. Laterally inclined flange along dorsal edge of surangular for articulation with lateral process of lateral quadrate condyle absent (0) or present (1 [5]).

122. Coossification of angular and surangular absent (0) or present (1) [17].

123. Angular exposed almost to end of mandible in lateral view, reaches or almost reaches articular (0) or excluded from posterior end; angular suture turns ventrally and meets ventral border of mandible rostral to glenoid (1) [12].

124. External mandibular fenestra present (0) or absent (1) [8].

125. External mandibular fenestra oval (0) or subdivided by a spinous rostral process of the surangular (1).

126. External mandibular fenestra not rostrally displaced (sits beneath orbit) (0) or rostrally displaced (sits anterior to orbit) (1) [4].

127. Internal mandibular fenestra small and slit-like (0) or large and rounded (1) [13].

128. Splenial not widely exposed on lateral surface of mandible (0) or exposed as a broad triangle between dentary and angular on lateral surface of mandible (1) [5].

129. Coronoid ossification large (0) or only a thin splint (1) or absent (2) [5].

130. Articular without elongate, slender medial, posteromedial, or mediodorsal process from retroarticular process (0) or with process (1) [5].

131. Mandibular articulation surface as long as distal end of quadrate (0) or twice or more as long as quadrate surface, allowing anteroposterior movement of mandible (1) [5].

132. Retroarticular process short, stout (0) or elongate and slender (1) [5].

133. Retroarticular process points caudally (0) or curves gently dorsocaudally (1) [12].

134. Premaxilla toothed (0) or edentulous (1) [5].

135. Premaxillary teeth serrated (0) or unserrated (1) [11].

136. Carinae of premaxillary teeth: present and not offset (0) or both carinae lingually located so that tooth has a D-shaped cross-section (1) or distal carina not offset but mesial carina mesially offset (2) or carinae absent (3) (modified from reference 5).

137. Second premaxillary tooth is not (0) or is markedly larger than third and fourth premaxillary teeth (1) [13].

138. Premaxilla without (0) or with first tooth incisiform and extremely enlarged (1) [3].

139. Premaxillary teeth subequal in size to (0) or much smaller than (1) the maxillary teeth [9].

140. Maxilla toothed (0) or edentulous (1) [5].

141. Maxillary and dentary teeth *ziphodont (pointed, recurved, and labiolingually flattened*), with crowns in middle of tooth row more than twice as high as the basal mesiolateral width (0) or lanceolate and subsymmetrical (1) or conical (2) or *ziphodont*, with crowns in middle of tooth row less than twice as high as the basal mesiolateral width (3) (reworded from reference 4).

142. Dentary fully toothed (0) or only with teeth rostrally (1) [12].

143. Longitudinal groove on labial surface of maxillary and dentary tooth crowns (venom groove of Gong et al. 2010) absent (0) or present (1) [3].

144. Maxillary and dentary teeth serrated (0) or some without serrations anteriorly (1) or all without serrations (2) [5].

145. Dentary and maxillary tooth size: large (usually, maxillary tooth crown height > 1/5 height of snout, and dentary tooth crown height > 1/2 height of dentary ramus) (1) or small (usually, maxillary tooth crown height < 1/6 height of snout, and dentary tooth crown height < 1/2 height of dentary ramus) (2) or miniscule (usually, maxillary tooth crown height < 1/10 height of snout, dentary tooth crown height < 1/3 height of dentary ramus) (3) [3].

In previous versions of this matrix [4,8], the character for tooth size was combined with number of teeth. These are actually two different characters. Tooth size can be estimated from the number and size of alveoli if teeth are missing or if the tooth-bearing bone only has teeth rostrally. For example, according to personal observations of myriad specimens by Phil Senter (1998 – 2010), taxa exhibiting state 1 tend to have alveoli large enough for < 20 teeth in a fully toothed maxilla or dentary (e.g. Tyrannosauroidea, Eudromaeosauria), taxa exhibiting state 3 tend to have alveoli small enough for > 50 teeth in a fully toothed maxilla or dentary (e.g. *Shuvuuia*, *Buitreraptor*), and taxa exhibiting state 2 tend to have alveoli of intermediate size (e.g. most Troodontidae).

146. Dentary toothed (0) or edentulous (1) (character state previously used, but newly used in reference 3 as its own character).

147. Dentary dentition continues cranially to tip of dentary (0) or terminates before reaching dentary tip (1) [4].

148. Dentary teeth do not (0) or do increase in size anteriorly (1) [12].

149. Dentary teeth evenly spaced (0) or anterior dentary teeth smaller, more numerous, and more closely appressed than those in middle of tooth row (1) or anterior dentary teeth more widely spaced than those in middle of tooth row (2) (modified from reference 5).

150. Serration denticles large (0) or small (1) [5]. This difference is quantified in reference 19.

151. Approximately the same number of denticles per 5 mm on mesial keels of teeth as on distal keels (0) or markedly more denticles per 5 mm on mesial keels (1) [10].

152. Serrations simple, denticles convex (0) or distal and often mesial edges of teeth with hooked denticles that point toward the tip of the crown (1) [5].

153. Teeth constricted between root and crown (0) or root and crown confluent (1) [5].

154. Roots of dentary and maxillary teeth mediolaterally compressed (0) or circular in cross-section (1) [12].

155. Maxillary and dentary teeth separated by interdental septa (0) or in on open groove (1) (modified from reference 9).

156. Number of cervical vertebrae: 10 (0) or 12 or more (1) [5].

157. Axial neural spine flared transversely (0) or compressed mediolaterally (1) [5].

158. Axial epipophyses absent or poorly developed, not extending past posterior rim of postzygopophyses (0) or large and posteriorly directed, extend beyond postzygapophyses (1) [5].

159. Cervical neural spines anteroposteriorly long and dorsoventrally tall (0) or anteroposteriorly short, dorsoventrally low, and centered on neural arch, giving arch an “X” shape in dorsal view (1) or anteroposteriorly short and dorsoventrally tall (2) or anteroposteriorly long and dorsoventrally short (3) (modified from reference 5).

160. Epipophyses of cervical vertebrae placed distally on postzygapophyses, above postzygapophyseal facets (0) or placed proximally, proximal to postzygapophyseal facets (1) [5].

161. Cervical prezygapophyses unflexed (0) or flexed (1) [9].

162. Anterior cervical centra subcircular or square in anterior view (0) or distinctly wider than high, kidney shaped (1) [5].

163. Anterior cervical centra level with or shorter than posterior extent of neural arch (0) or centra extending beyond posterior limit of neural arch (1) [5].

164. Shortening of cervical centra to less than the height of the centrum: absent (0) or present (1) [20].

165. Carotid process on posterior cervical vertebrae absent (0) or present (1) [5].

166. Cervical centra with one pair of pneumatic openings (0) or with two pairs of pneumatic openings (1) [5].

167. Cervical ribs unfused to cervical vertebrae (0) or fused to cervical vertebrae (1) [17].

168. Shaft of cervical ribs slender and longer than vertebra to which they articulate (0) or broad and shorter than vertebra (1) [5].

169. Length of mid-cervical centra approximately the same as dorsal centra (0) or markedly longer than dorsal centra (1) [10].

170. Cervical and anterior trunk vertebrae amphiplatyan (0) or opisthocoelous (1) [5].

171. Posterior dorsal centra > 1.2 x taller than long (0) or height subequal or a little less than length (1) or length > 2x height (2) (modified from reference 10).

172. Anterior trunk vertebrae without prominent hypapophyses (0) or with large hypapophyses (1) [5].

173. Middle and posterior dorsal vertebrae not pneumatic (0) or pneumatic (1) [4].

174. Parapophyses of posterior trunk vertebrae flush with neural arch (0) or distinctly projected on pedicels (1) [5].

175. Posterior dorsal neural spines > 1.5 x taller than long (0) or height < 1.5 x length (1) [10].

176. Neural spines on posterior dorsal vertebrae in lateral view rectangular or square (0) or anteroposteriorly expanded distally, fan-shaped (1) or shaped like partial fan, with posterior corner of fan present but anterior corner absent (2) (modified from reference 12).

177. Hyposphene-hypantrum articulations in trunk vertebrae absent (0) or present (1) [5].

178. Postzygapophyses of middle and posterior dorsal vertebrae do not extend posterior to centrum (0) or do (1) [6].

179. Zygapophyses of trunk vertebrae abutting one another above neural canal, opposite hyposphenes meet to form lamina (0), or zygapophyses placed lateral to neural canal and separated by groove for interspinous ligaments, hyposphenes separated (1) [5].

180. Transverse processes of anterior dorsal vertebrae long and thin (0) or short, wide, and only slightly inclined (1) [5].

181. Neural spines of dorsal vertebrae not expanded distally (0) or expanded to form ‘spine table’ (1) [5].

182. Number of sacral vertebrae: 5 (0) or 6 (1) or 7 or more (2) (modified from reference 5).

183. Sacral vertebrae with unfused zygapophyses (0) or with fused zygapophyses forming a sinuous ridge in dorsal view (1) [5].

184. Ventral surface of posterior sacral centra gently rounded, convex (0) or ventrally flattened, sometimes with shallow sulcus (1) or centrum strongly constricted transversely, ventral surface keeled (2) [5].

185. Pleurocoels absent on sacral vertebrae (0) or present on anterior sacrals only (1) or present on all sacrals (2) [5].

186. Last sacral centrum with flat posterior articulation surface, caudal centra amphiplatyan (0) or convex articulation surface, caudal centra procoelous (1) (modified from reference 5).

187. More than 30 caudal vertebrae (0) or 21-30 caudal vertebrae (1) or < 10 caudal vertebrae, followed by pygostyle (2) or 11 – 20 vertebrae (3) [4].

188. Anterior caudal vertebrae without pneumatopores (0) or with pneumatopores (1) [21].

189. Anterior caudal centra craniocaudally short relative to height (0) or at least twice as long as tall (2) [3].

190. Anterior caudal centra tall, oval in cross section (0) or with box-like centra in caudals 1 – 5 (1) or anterior caudal centra laterally compressed with ventral keel (2) [5].

191. Caudal vertebrae without distinct transition point (0) or with type 1 transition point (term introduced by Senter (2011)): abrupt change in vertebral and hemal arch morphology characterized by prezygapophyseal elongation, loss of transverse processes, and change of hemal arch shape into an inverted T (1) or with type 2 transition point (term introduced in reference 3): abrupt change in vertebral and hemal arch morphology characterized by extreme reduction in neural spine height, loss of transverse processes, elongation of centra, and change of hemal arch shape into an inverted T (2) [3]. State 0 was formerly state 1, and former state 0 is now separated into two states: current states 1 and 2; state 1 lacks central elongation and sudden reduction in neural spines, and state 2 lacks prezygapophyseal elongation except in most Dromaeosauridae.

192. Transition point in caudal series begins distal to the 10th caudal (0) or between 7th and 10th caudal vertebra (1) or proximal to the 7th caudal vertebra (2) [22].

193. Lengths of mid-caudal centra subequal to or less than those of proximal caudal centra (0) or > twice as long as proximal caudal centra (1) or between 1.3x and 2x as long as proximal centra (2) (modified from reference 4).

194. Midline sulcus in center of neural arch of distal caudals absent (0) or present (1) (modified from reference 5).

195. Prezygapophyses of distal caudal vertebrae between 1/3 and whole centrum length (0) or with *bifurcated and* extremely long extensions of the prezygapophyses (up to 10 vertebral segments long in some taxa) (1) or strongly reduced as in *Archaeopteryx lithographica* (2) *extended in length but not as long as in state 1 (prezygapophyseal length approximately 1 to 1 ½ centrum lengths) and not bifurcated (3)* (from reference 5, *with state 3 newly added*).

196. Caudotheca (term introduced in reference 3: network of elongated prezygapophyses and hemal arches in the tails of dromaeosaurids), if present, reaches caudal vertebra 3 or 4; anteriormost vertebra with elongated prezygapophyses is caudal 4, 5, or 6 (0) or reaches no further forward than caudal 6; anteriormost vertebra with elongated prezygapophyses is caudal 8, 9, or 10 (1) [3].

*197*. Anteriormost hemal arches > 1.5 x longer than associated centra (0) or < 1.5 x as long as centra (1) [10].

198. Extreme elongation of anteriormost hemal arches (to > 4x dorsoventral height of associated centra): absent (0) or present (1) [3].

199. Proximal end of chevrons of proximal caudals short anteroposteriorly, shaft proximodistally elongate (0) or proximal end elongate anteroposteriorly, flattened and plate-like (1) [5].

200. Distal caudal chevrons are simple (0) or anteriorly bifurcate (1) [5]. Formerly, this character was listed with a state 2: chevrons bifurcated anteriorly and posteriorly. However, this can vary along the length of the tail even within a single specimen (e.g. [23]).

201. Distal chevrons straight or L-shaped in lateral view (0) or upside-down T-shaped (1) [4].

202. Ossified uncinate processes absent (0) or present (1) [5].

203. Ossified ventral rib segments absent (0) or present (1) [5].

204. Lateral gastralial segment shorter than medial one in each arch (0) or distal segment longer than proximal segment (1) [5].

205. Ossified sternum demonstrably absent (0) or present (1) [8].

206. Ossified sternal plates separate in adults (0) or fused (1) [5].

207. Sternum without distinct lateral xiphoid process posterior to costal margin (0) or with lateral xiphoid process (1) [5].

208. Anterior edge of sternum grooved for reception of coracoids (0) or sternum without grooves (1) [5].

209. Articular facet of coracoid on sternum: anterolateral or more lateral than anterior (0); almost anterior (1) [5].

210. Angle between furcular arms > 80^o^ (0) or < 60^o^ (1) [4].

211. Hypocleidium on furcula absent or very small (0) or a prominent prong (1) (modified from reference 5).

212. Wide distal expansion of scapula absent (0) or present such that scapular blade is an elongate triangle (1) or present such that scapular blade is hatchet-shaped (sudden widening about midway up the anterior edge, but widened only distally along posterior edge) (2) or present such that scapular blade is distally paddle-shaped, with rounded rather than pointed corners at the distal expansion (3) (modified from reference 4).

213. Acromion margin of scapula continuous with blade (0) or anterior edge laterally everted (1) [5].

214. Acromion process does not match any of the following descriptions: (0) rectangular with its dorsal edge forming a 90^o^ angle with the dorsal edge of the scapular blade (1) or a quarter-circle in shape (2) or reduced and does not contact coracoid (3) (modified from reference 4).

215. Flange on supraglenoid buttress on scapula absent (0) or present (1) [12].

216. Glenoid fossa without (0) or with extension of glenoid floor onto external surface of scapula (the surface opposite the costal surface) (1) [4].

217. Scapula and coracoid separate (0) or fused into scapulocoracoid (1) [5].

218. Scapulocoracoid dorsal margin: pronounced notch between the acromion process and the coracoid (0) or margin smooth (1) [9].

219. Scapula and coracoid form a continuous arc in posterior and anterior views (0) or coracoid inflected medially, scapulocoracoid ‘L’ shaped when viewed along edge of scapular blade (1) [5].

220. Coracoid in lateral view subcircular, with shallow ventral blade (0) or subquadrangular with extensive ventral blade (1) or shallow ventral blade with elongate posteroventral process (2) or subtriangular (proximal end constricted, distal end wide) (3) [4].

221. Posterior edge of coracoid not or shallowly indented below glenoid (0), or posterior edge of coracoid deeply notched just ventral to glenoid, glenoid lip everted (1) [12].

222. Anterior surface of coracoid ventral to glenoid fossa unexpanded (0) or anterior edge of coracoid expanded, forms triangular subglenoid fossa bounded laterally by coracoid tuber (1) [5].

223. Acrocoracoid process absent (0) or present (1) [4].

224. Scapula longer than humerus (0) or humerus longer than scapula (1) [5].

225. Humeral length is half femoral length or less (0) or shorter than femur but more than half femoral length (1) or longer than femur (2) [10].

226. Length of humeral shaft between deltopectoral crest and distal condyles < 4.5 x shaft diameter (0) or > 4.5 x shaft diameter (1) [4].

227. Deltopectoral crest large and distinct, proximal end of humerus quadrangular in anterior view (0) or deltopectoral crest less pronounced, forming an arc rather than being quadrangular (1) or deltopectoral crest very weakly developed, proximal end of humerus with rounded edges (2) or deltopectoral crest extremely long (3) or proximal end of humerus extremely broad, triangular in anterior view (4) [5].

228. Distal humerus with small or no medial epicondyle (0) or with large medial epicondyle, medial condyle centered on distal end (1) [12].

229. Lateral epicondyle of humerus not expanded laterally (0) or expanded laterally (1) [4].

230. Distal humeral condyles on distal end (0) or on anterior surface and are two distinct condyles (1) or on anterior surface and are a single condyle (2) (modified from reference 12).

231. Width of distal humeral expansion < 1/3 humeral length (0) or > 1/3 humeral length (1) [4].

232. Length of radius < 1/3 femoral length (0) or between 1/3 and 2/3 femoral length (1) or between 2/3 and 1x femoral length (2) or > femoral length (3) [4].

233. Antebrachial bones stout (radial shaft length < 8x diameter, ulnar shaft length < 6x diameter) (0) or gracile (radial shaft length > 8x diameter, ulnar shaft length > 6x diameter (1) [3].

234. Radial diameter > 0.5 x ulnar diameter (0) or < 0.5 x (1) [10].

235. Radius straight (0) or shallowly sigmoid so that distal half is bowed away from ulna (1) [3].

236. Radius and ulna well separated (0) or with distinct adherence or syndesmosis distally (1) [12].

237. UIna not bowed away from radius (0), or bowed away from radius (1) [24].

238. Olecranon process weakly developed (0) or distinct and large but not hypertrophied (1) or hypertrophied (2) [4].

239. Proximal surface of ulna a single continuous articular facet (0) or divided into two distinct fossae separated by a median ridge (1) [5].

240. Distal articular surface of ulna flat (0) or convex, semilunate surface (1) [5].

241. Lateral proximal carpal (ulnare?) quadrangular (0) or triangular in proximal view (1) [5]. The homology of the carpal elements of coelurosaurs is unclear [25] but the large, triangular lateral element of some taxa most likely corresponds to the lateral proximal carpal of basal tetanurans.

242. Two distal carpals in contact with metacarpals, one covering the base of metacarpal I (and perhaps contacting metacarpal II) the other covering the base of metacarpal II (distal carpals 1 and 2 unfused) (0) or a single distal carpal capping metacarpals I and II (distal carpals 1 and 2 fused) (1) (modified from reference 5).

243. Distal carpals 1+2 flattish (0) or shallowly convex (0) or strongly semilunate in shape (2) (modified from reference 10).

244. Distal carpals 1+2 well developed, covering all of proximal ends of metacarpals I and II (0) or small, cover about half of base of metacarpals I and II (1) or cover bases of all metacarpals (2) [4].

245. Distal carpals not fused to metacarpals (0) or fused to metacarpals, forming carpometacarpus (1) [5].

246. Extensor pits on dorsal surface of distal end of metacarpals: pronounced (0) or shallow or absent (1) [11].

247. Metacarpal I half or less than half the length of metacarpal II, and longer proximodistally than wide transversely (0) or subequal in length to metacarpal II (1) or very short and wider transversely than long proximodistally (2) or about two-thirds the length of metacarpal II (3) [5].

248. Distal end of metacarpal I medially (0) or laterally rotated (1) (Pérez-Moreno et al. 1994).

249. Metacarpal II > 1/3 humeral length (0) or < 1/3 humeral length (1) [4].

250. Distal articular ends of metacarpals I + II ginglymoid (0) or rounded, smooth (1) [12].

251. Metacarpal III distally not ginglymoid (0) or ginglymoid (1) [4].

252. Length of manual digit II (including metacarpal) less than 1.25 x femoral length (0) or > 1.25 x femoral length (1) [4].

253. Medial side of metacarpal II: expanded proximally (0), not expanded (1) [9].

254. Metacarpals II and III are not (0) or are appressed for their entire lengths (1) [4].

255. Proximal end of metacarpal III is not (0) or is mainly palmar to that of metacarpal II (1) [9].

256. Metacarpal III > 0.75 x length of metacarpal II (0) or < 0.75 x (1) [8].

257. Medial ligament pits of manual phalanges deep (0) or shallow (1) [26].

258. With fingers extended, tip of ungual I does not extend past flexor tubercle of ungual II (0) or extends past flexor tubercle of ungual II (1) [4] (coded “?” for “not applicable” if the ungual of digit I is hugely enlarged).

259. Manual phalanx I-1 straight (0) or bowed (palmar surface concave) (1) [10].

260. Manual phalanx I-1 longer than metacarpal II (0) or shorter or subequal in length (1) [18].

261. Length of metacarpal II < length of metacarpal I + phalanx I-1 (0) or > (1) [10].

262. Shaft diameter of manual phalanx I-1 less (0) or greater (1) than shaft diameter of radius [12].

263. Length of manual phalanx II-2 < 1.2 x length of phalanx II-1 (0) or > 1.2 x (1) [10].

264. Manual phalanx II-1 without (0) or with pronounced widening in the plane of the hand such that a posterior flange is present in full or incipiently (1) (reworded from reference 10).

265. Combined lengths of manual phalanges II-1 and II-2 > length of metacarpal II +carpus (0) or < length of metacarpal II +carpus (1) [4].

266. Manual phalanx II-1 shorter than I-1 (0) or longer (1) [4].

267. Length of manual phalanx II-1 < 2 x length of III-1 (0) or > 2 x length of III-1 (1) [10].

268. Length of manual phalanx II-2 < 2 x length of II-1 (0) or > 2 x (1) [4].

269. With fingers extended, tip of ungual III extends no further distally than flexor tubercle of ungual II (0) or extends further (1) [4].

270. Diameter of non-ungual phalanges of manual digit III > 0.5 x diameter of non-ungual phalanges of digit II (0) or < 0.5 x (1) [4].

271. Third manual digit present, phalanges present (0) or reduced to no more than metacarpal splint (1) [5].

272. Manual digit III with four phalanges (0) or less than four phalanges (1) [4].

273. Manual phalanx III-3 markedly shorter than combined lengths of phalanges III-1 and III-2 (0), subequal in length to their combined lengths (1), or markedly longer (2) [4].

274. Length of manual phalanx III-1 < 2 x length of phalanx III-2 (0) or > 2 x (1) [10].

275. Length of manual unguals distal to flexor tubercle is much greater than height of articular facet (0) or is not (1) [8].

276. Flexor tubercles of manual unguals proximal (0) or displaced distally from articular end (1) or proximodistally elongated with proximal end close to articular facet (2) [4]

277. Flexor tubercles of manual unguals > 1/3 x height of articular facet (0) or < 1/3 (1) (modified from reference 11).

278. Unguals on first two digits generally similar in size (0) or digit I bearing large ungual and unguals of other digits distinctly smaller (1) [5].

279. With proximal articular surface of ungual oriented vertically, dorsal surface of manual ungual I does not (0) or does arch higher than level of dorsal extremity of proximal articular surface (1) [10].

280. With proximal articular surface of ungual oriented vertically, dorsal surface of manual ungual II does not (0) or does arch higher than level of dorsal extremity of proximal articular surface [10].

281. Manual ungual I strongly curved (0), weakly curved (1), or straight (2) [4].

282. Manual unguals II and III strongly curved (0), weakly curved, (1), or straight (2) [4].

283. Pronounced proximodorsal ‘lip’ (transverse ridge immediately dorsal to the articulating surface) on first manual ungual absent (0) or present (1) [4].

284. Pronounced proximodorsal “lip” on manual unguals II and III absent (0) or present (1) [4].

285. Manual ungual III smaller than ungual II (0) or approximately the same size (1) [4].

286. Preacetabular portion of ilium parasagittal (0) moderately laterally flaring (1) strongly laterally flaring (2) [12].

287. Anterior end of ilium gently rounded or straight (0) or anterior end strongly curved (1) or pointed at anterodorsal corner (2) or with notch at anterodorsal corner (3) (modified from reference 5).

288. Ventral edge of anterior ala of ilium straight or gently curved (0) or ventral edge hooked anteriorly (1) [5].

289. Anteroventral corner of preacetabular ala of ilium is not (0) or is posteriorly displaced (1) [3]. (illustrated in reference 3, fig. S1i)

290. Arching of preacetabular iliac blade above height of postacetabular blade absent or small (0) or extreme (1) [4].

291. Ridge bordering cuppedicus fossa extends far posteriorly and is confluent or almost confluent with acetabular rim (0) or ridge terminates rostral to acetabulum or curves ventrally onto anterior end of pubic peduncle (1) [22].

292. Cuppedicus fossa deep, ventrally concave (0) or fossa shallow or flat, with no lateral overhang (1) or absent (2) [5].

293. Preacetabular part of ilium roughly as long as postacetabular part of ilium (0) or preacetabular portion of ilium markedly longer (more than 2/3 of total ilium length) than postacetabular part (1) [5].

294. Ventral edge of postacetabular ala of ilium horizontal and high (approximately the level of the dorsal edge of the acetabulum) (0) or downturned, so that tip is approximately level with ventral edges of the peduncles of the ilium (1) or downturned, so that tip is far ventral to the level of the ventral edges of the peduncles (2) [3]. (illustrated in reference 3, fig. S1j)

295. Postacetabular ala of ilium in lateral view squared (0) or sharply acuminate (1) [5].

296. Brevis fossa shelf-like (0) or deeply concave with lateral overhang (1) [5].

297. Brevis fossa poorly developed adjacent to ischial peduncle and without lateral overhang, medial edge of brevis fossa visible in lateral view (0), or fossa well developed along full length of postacetabular blade, lateral overhang extends along full length of fossa, medial edge completely covered in lateral view (1) [12].

298. Dorsal margin of postacetabular iliac blade straight or convex (0) or concave (1) [22].

299. Postacetabular blades of ilia in dorsal view parallel (0) or diverge posteriorly (1) [5].

300. Pubic peduncle of ilium craniocaudally longer (0) or shorter (1) than ischial peduncle of ilium [4].

*301. In lateral view, distal (pubic) surface of public peduncle of ilium slanted so that it faces somewhat cranially (0) or subhorizontal (1) or slanted so that it faces somewhat caudally (2) (new character)*

302. Vertical ridge on ilium, dorsal to acetabulum, absent (0) or present (1) [11]

303. Supraacetabular crest on ilium as a separate process from antitrochanter, forms “hood” over femoral head present (0) reduced, not forming hood (1) or absent (2) [5].

304. Antitrochanter posterior to acetabulum absent or poorly developed (0) or prominent (1) [5].

305. Length of pubis not reduced (similar to that of femur) (0) or strongly reduced (1) [8].

306. Pubis propubic (0) or pubis vertical (1) or pubis moderately posteriorly oriented (2) or pubis fully posteriorly oriented (opisthopubic) (3) [5]. The oviraptorid condition, in which the proximal end of the pubis is vertical and the distal end curves anteriorly, is considered to be state 1. *This is determinable even for isolated pubes. For example, for taxa in which the pubic surface of the pubic peduncle of the ilium is horizontal (Compsognathidae, Paraves), if the shaft of the pubis is perpendicular to the iliac surface of the pubis, then the pubis is vertical. Previous authors have considered the pubis to be retroverted in* Velociraptor *and other eudromaeosaurs [27]. However, in Paraves the pubic surface of the pubic peduncle of the ilium is horizontal [23,27–32]. This means that if the iliac surface of the pubis is perpendicular to the pubic shaft, then the pubic shaft was vertical. PS has confirmed this in the dromaeosaurids* Rahonavis *and* Unenlagia *by manual articulation of originally disarticulated pelves. The iliac surface of the pubis is perpendicular to the pubic shaft in the eudromaeosaurs* Velociraptor *[33] and* Achillobator *[29] and the basal microraptorian* Tianyuraptor *[34]. Their pubes were therefore vertical, not retroverted. The retroversion of their pubes in articulated specimens [27,33,34] is due to disarticulation of the pubes from the ilia and subsequent rotation of the pubes. A similar situation is present in* Archaeopteryx*. Its pubes have become disarticulated from the ilia and rotated into a retroverted position in most specimens [31,35,36]; the pubes are vertical in the one specimen of* Archaeopteryx *in which they retain articulation with the ilia [37]. Advanced microraptorians are the only dromaeosaurids in which the pubes are retroverted. In advanced microraptorians the pubic shafts are at a strong angle to the iliac surface of the pubis [38,39], and an articulated specimen demonstrates that this causes their pubes to be retroverted [39].*

*307. Pubic tubercle (sensu reference 20) not prominent or not as in sate 1 (0) or prominent and laterally compressed and situated on cranial surface of iliac peduncle (1) (new character).*

308. Pubic shaft straight (0) or distal end curves anteriorly, anterior surface of shaft concave in lateral view (1) or anterior surface of shaft convex in lateral view (2) or pubis strongly kinked at midshaft, caudally displacing distal half (3) (modified from reference 4).

309. Lateral tubercle about halfway down pubic shaft absent (0) or present (1) [40].

310. Pubic apron present (0) or absent (1) [3].

311. Pubic apron extends medially from middle of cylindrical pubic shaft (0) or shelf extends medially from anterior edge of anteroposteriorly flattened shaft (1) [5].

312. Pubic apron about half of pubic shaft length (0) or less than 1/3 of shaft length (1) [5].

313. Length of pubic boot < 30% length of pubis (0) or > 40% (1) [4]

314. Hyperenlargement of pubic boot to > 2/3 length of pubis: absent (0) or present (1) [20].

315. Distal end of pubis: with pubic boot projecting anteriorly and posteriorly (0) or with posterior process but little or no anterior process (1) or with no anteroposterior projections (2) or without boot but slightly expanded and spatulate in lateral view (3) (modified from reference 5).

316. Ischium more than 70% (0) or 70% or less of pubis length (1) (modified from reference 5).

317. Shafts of pubis and ischium not in contact (0) or appressed (1) [3].

318. Ilium and ischium articulation flat or slightly concavo-convex (0) or ilium with process projecting into socket in ischium (1) [12].

319. Ischial shaft rodlike (0) or flat, platelike (1) [22].

320. Lateral face of ischial shaft flat (or round in rodlike ischia) (0) or laterally concave (1) or with longitudinal ridge dividing lateral surface into anterior and posterior parts (2) or with longitudinal groove *(3)* (modified from reference 22).

321. Thickness of shaft of ischium (distal to obturator process if one is present): unreduced (0), reduced, slenderer than the pubic shaft (1) (modified from reference 9).

322. Shaft of ischium straight in lateral view (0) or ventrodistal end curved anteriorly (1) or curved dorsally (posterodorsally concave) (2) [6].

323. Posterior edge of ischium without (0) or with prominent proximodorsal prong (1) [4]

324. Dorsal process along caudal edge of ischial shaft, about halfway down the shaft: absent (0) or present and proximodistally elongate (1) present as a small, almost pointed tuber (modified from reference 28).

325. Semicircular scar on posterior part of the proximal end of the ischium, absent (0) or present (1) [5].

326. Proximoventral plate of ischium fully expressed (0) or reduced to obturator process (1) or completely absent, including obturator process (2) [3].

327. Obturator process proximal in position (0) or distally displaced (1) (modified from reference 5).

328. Obturator process does not contact pubis (0) or contacts pubis (1) [5].

329. Obturator process not as in state 1 (0) or in lateral view is elongate and forms a strongly acute angle that points perpendicular to ischial shaft (1) (modified from reference 10).

330. Obturator process does not (0) or does reach tip of ischium (1) [10].

331. Ventral notch between the distal portion of the obturator process and the shaft of the ischium: present (0), absent (1) [9].

332. Distal ends of ischia form symphysis (0) or approach one another but do not form symphysis (1) or widely separated (2) [5].

333. Ischial boot (expanded distal end) present (0) or absent (1) [5].

334. In adult, femur longer than tibia (0) or shorter (1) [10].

335. Femoral head without fovea capitis (for attachment of capital ligament) (0) or circular fovea present in center of medial surface of head (1) [5].

336. Lesser and greater trochanters unfused (0) or fused (1) [4].

337. Tip of lesser trochanter below level of femoral head (0) or level with femoral head (1) [9].

338. Lesser trochanter of femur alariform (0) or cylindrical in cross section (1) [5].

339. Vertical ridge on lesser trochanter present (0) or absent (1) [12].

340. Accessory trochanteric crest distal to lesser trochanter absent (0) or present (1) [5].

341. Posterior trochanter (sensu reference 23) absent or represented only by rugose area (0) or posterior trochanter distinctly raised from shaft, mound-like (1).

342. Lateral trochanter (term introduced by Senter [8}) for anteriorly projecting lateral flange on proximal end of femur, immediately distal to lesser trochanter, as in *Caudipteryx* and *Microvenator*) absent (0) or present (1) [8].

343. Fourth trochanter on femur present (0) or absent (1) [5].

344. Anterior surface of femur proximal to medial distal condyle without longitudinal crest (0) or crest present extending proximally from medial condyle on anterior surface of shaft (1) [5].

345. Popliteal fossa on distal end of femur open distally (0) or closed off distally by contact between distal condyles (1) [5].

346. Proximolateral (fibular) condyle of the tibia, development in proximal view: bulge from the main surface of the tibia (0), conspicuous narrowing between the body of the condyle and the main body of the tibia (1) [9].

347. Medial cnemial crest absent (0) or present on proximal end of tibia (1) [5].

348. Fibula reaches proximal tarsals (0) or short, tapering distally, and not in contact with proximal tarsals (1) [5].

349. Medial surface of proximal end of fibula concave along long axis (0) or flat (1) [5].

350. Deep oval fossa on medial surface of fibula near proximal end absent (0) or present (1) [5].

351. Ascending process of the astragalus tall and broad, covering most of anterior surface of distal end of tibia (0) or process short, covering only lateral half of anterior surface of tibia (1) or ascending process tall with medial notch that restricts it to lateral side of anterior face of distal tibia (2) [5].

352. Ascending process of astragalus confluent with condylar portion (0) or separated by transverse groove or fossa across base (1) [5].

353. Distal end of tibia and astragalus without distinct condyles (0) or with distinct condyles separated by prominent tendinal groove on anterior surface (1) [5].

354. Astragalus and calcaneum separate from tibia (0) or fused to each other and to the tibia in late ontogeny (1) [5].

355. Distal tarsals separate, not fused to metatarsals (0) or form metatarsal cap with intercondylar prominence that fuses to metatarsal early in postnatal ontogeny (1) [5].

356. Metatarsus less than half length of femur (0) or more than half femoral length (1) [4].

357. Metatarsal cross-sectional proportions: subequal or wider mediolaterally than craniocaudally at midshaft (0), deeper craniocaudally than mediolaterally at midshaft (1) [9].

358. Shafts of metatarsals distally divergent and not appressed (0) or appressed (1) or straight and not appressed (2) (modified from reference 4).

359. Marked mediolateral decrease in transverse width of metatarsus distally, absent (0) or present (1) [4].

360. Length of metatarsus < 3.5x transverse midshaft diameter (0) or 3.5x – 8x midshaft diameter (1) or > 8x midshaft diameter (2) [4].

361. Metatarsals not co-ossified (0) or co-ossification of metatarsals begins proximally (1) or distally (2) [5].

362. Metatarsal I present (0) or absent (1) (modified from reference 5).

363. Metatarsal I articulates at middle of metatarsal II (0) or metatarsal I attaches to distal quarter of metatarsal II (1) or metatarsal I articulates with metatarsal II near its proximal end (2) (modified from reference 5).

364. Shaft of metatarsal I: reduced to a splint (0) or unreduced (1) (reworded from character 203 of reference 8).

365. Distal end of metatarsal I reduced in size relative to distal ends of other metatarsals (0) or comparable in size to distal ends of other metatarsals (1) [4].

366. Strong bevelling of distal end of metatarsal II (medial condyle strongly proximally offset from lateral condyle) absent (0) or present (1) [3].

367. Distal end of metatarsal II smooth, not ginglymoid (0) or with developed ginglymus (1) [5].

368. Distal end of metatarsal III smooth, not ginglymoid (0) or with developed ginglymus (1) [5].

369. In anterior view, metatarsal III not pinched (0) or pinched proximally (1) or pinched both proximally and through midshaft (2) [4].

370. Anteroproximal contact between metatarsals II and IV absent (0) or present (1) [4].

371. Metatarsal III contributes to proximal surface of metatarsus (0) or does not (1) [3].

372. Shaft of MT IV round or thicker dorsoventrally than wide in cross section (0) or shaft of MT IV mediolaterally widened and flat in cross section (1) [5].

373. Proximal end of metatarsal IV curls around plantar side of proximal end of metatarsal III (0) or doesn’t (1) [41].

374. Large, longitudinal flange along caudal or lateral face of metatarsal IV absent (0) or present (1) [42].

375. Foot symmetrical (0) or asymmetrical with slender metatarsal II and very robust metatarsal IV (1) [12].

376. Length of metatarsal V > 0.5 x length of metatarsal IV (0) or < 0.5 x (1) [10].

377. Plantar surface of hallux faces posteriorly (0) or hallux reoriented so that plantar surface faces medially or anteriorly (1) [10]. This is diagnosable by the degree of torsion in metatarsal I, even in disarticulated specimens [43].

378. Pedal phalanx II-1 longer (0) or shorter (1) than pedal phalanx IV-1 [10].

379. Pedal phalanx II-1 without dorsal extension of distal condyles (0) or with extension (1) [4].

380. Total length of pedal phalanx II-1 much greater than 2 x length of distal condylar eminence (0) or only slightly greater than 2 x (1) [3].

381. Length of pedal phalanx II-2 between 0.6 x and 1 x length of phalanx II-1 (0), < 0.6 x, or (1) > 1 x (2) [10].

382. Total length of pedal phalanx II-2 (not counting posteroventral lip, if any) > 2 x length of distal condylar eminence (0) or < 2 x (1) [10].

383. Pedal phalanx II-2 with distinct shaft or waist between proximal cotyles and distal condylar eminence (0) or shaft eliminated by extreme shortening of this phalanx (1) [3].

384. Pedal phalanx II-2 without posteroventral lip or keel (0) with transversely wide posteroventral lip (1) with transversely narrow posteroventral keel (2) [22].

385. Difference in lengths of toes II and IV (exclusive of unguals) small (0) or extreme, with toe II shorter (1) [8].

386. Phalanges of pedal digit III not blocky (proximal phalanx length > 2x diameter) (0) or blocky (proximal phalanx length < 2x diameter) (1) [4].

387. Cross-section of pedal unguals II, III and IV triangular (0) or a vertical oval (1) [44].

388. Hallucal ungual reduced in size relative to other pedal unguals (0) or not reduced (1) [4].

389. Hallucal ungual weakly curved (0) or strongly curved (1) [4].

390. Ungual of pedal digit II: similar in size to that of III (0) enlarged, about 50% larger than pedal ungual III, and much longer than pedal phalanx II-1 (1) or enlarged but similar in length to pedal phalanx II-1 (2) (modified from reference 4).

391. Distinct notch demarcating proximal end of flexor tubercle of pedal ungual II in lateral view absent (0) or present (1) [8].

392. Pedal unguals II – IV (not counting ungual II if it is enlarged) straight or weakly curved (0), or strongly curved (1) [4].

393. Pedal unguals II – IV (not counting ungual II if it is enlarged) without (0) or with prominent flexor tubercles (1) [3].

394. Medial and lateral spurs on unguals II – IV: absent (0) or present (1) [3].

**References**

1. Vargas AO, Fallon JF (2005) Birds have dinosaur wings: the molecular evidence. J Exp Zool (Mol Dev Evol) 304B: 86-90.

2. Tamura K, Nomura N, Seki R, Yonei-Tamura Y, Yokoyama H (2011) Embryological evidence identifies wing digits in birds as digits 1, 2, and 3. Science 331: 753-757.

3. Senter P (2011) Using creation science to demonstrate evolution 2: morphological continuity within Dinosauria. J Evol Biol doi: 10.1111/j.1420-9101.2011.02349.x

4. Senter P (2007) A new look at the phylogeny of Coelurosauria. J Syst Palaeontol 5: 429-463.

5. Norell MA, Clark J M, Makovicky P J (2001) Phylogenetic relationships among coelurosaurian dinosaurs. In: Gauthier J, Gall LF, editors. New perspectives on the origin and evolution of birds. New Haven: Yale University Press. pp. 49-67..

6. Maryańska T, Osmólska H, Wolsan M (2002) Avialan status for Oviraptorosauria. Acta Palaeontol Pol 47: 97-116.

7. Chiappe LM, Norell MA, Clark JM (1998) The skull of a relative of the stem-group bird *Mononykus.* Nature 392: 275-278.

8. Senter P (2010) Using creation science to demonstrate evolution: application of a creationist method for visualizing gaps in the fossil record to a phylogenetic study of coelurosaurian dinosaurs. J Evol Biol 23: 1732-1743.

9. Holtz TR Jr, Molnar RE, Currie PJ (2004) Basal Tetanurae. In: Weishampel DP, Dodson P, Osmólska H, editors. The Dinosauria, Second Edition. Berkeley: University of California Press. pp. 71-100.

10. Senter P, Barsbold R, Britt BB, Burnham DA (2004) Systematics and evolution of Dromaeosauridae (Dinosauria: Theropoda). Bull Gunma Mus Nat. Hist 8: 1-20.

11. Rauhut OWM (2003) The interrelationships and evolution of basal theropod dinosaurs. Sp Pap Paleontol 69: 1-215.

12. Kirkland JI, Zanno LE, Samson SD, Clark JM, DeBlieux DD (2005) A primitive therizinosauroid dinosaur from the Early Cretaceous of Utah. Nature 435: 84-87.

13. Currie PJ (1995) New information on the anatomy and relationships of *Dromaeosaurus albertensis* (Dinosauria: Theropoda). J Vertebr Paleontol 15: 576-591.

14. Currie PJ (1985) Cranial anatomy of *Stenonychosaurus* (Saurischia, Theropoda) and its bearing on the origin of birds. Can J Ea Sci 22: 1643-1658.

15. Currie PJ, Zhao X (1993) A new troodontid (Dinosauria, Theropoda) braincase from the Dinosaur Park Formation (Campanian) of Alberta. Can J Ea Sci 30: 2231-2247.

16. Makovicky PJ, Sues H-D (1998) Anatomy and phylogenetic relationships of the theropod dinosaur *Microvenator celer* from the Lower Cretaceous of Montana. Am Mus Novit. 3240: 1-27.

17. Osmólska H, Currie PJ, Barsbold R (2004) Oviraptorosauria. In: Weishampel DP, Dodson P, Osmólska H, editors. The Dinosauria, Second Edition. Berkeley: University of California. pp. 1665-183.

18. Pérez-Moreno BP, Sanz JL, Buscalioni AD, Moratalla JJ, Ortega F, Rasskin-Gutman D (1994) A unique multitoothed ornithomimosaur from the Lower Cretaceous of Spain. Nature 370: 363-367.

19. Farlow JO, Brinkman DL, Abler WL, Currie PJ (1991) Size, shape, and serration density of theropod dinosaur lateral teeth. Mod Geol 16: 161-198.

20. Hutchinson JR (2001) The evolution of pelvic osteology and soft tissues on the line to extant birds (Neortnithes). Zool J Linn Soc 131: 123-168.

21. Holtz TR Jr (1998) A new phylogeny of the carnivorous dinosaurs. Gaia 68: 1100-1117.

22. Makovicky PJ, Apesteguía S, Angolín FL (2005) The earliest dromaeosaurid theropod from South America. Nature 347: 1007-1011.

23. Ostrom JH (1969) Osteology of *Deinonychus antirrhopus*, an unusual theropod from the Lower Cretaceous of Montana. Peab Mus Nat Hist Bull 30: 1-165.

24. Gauthier J (1986) Saurischian monophyly and the origin of birds. Mem Calif Acad Sci 8: 1-55.

25. Padian K, Chiappe LM (1998) The origin and early evolution of birds. Biol Rev 73: 1-42.

26. Clark JM, Maryańska T, Barsbold R (2004) Therizinosauroidea. In: Weishampel DB, Dodson P., Osmólska H, editors. The Dinosauria, Second Edition. Berkeley: University of California Press pp. 151-164.

27. Norell MA, Makovicky PJ (1997) Important features of the dromaeosaur skeleton: information from a new specimen. Am Mus Novit 3215: 1-28.

28. Forster CA, Sampson SD, Chiappe LM, Krause DW (1998) The theropod ancestry of birds: new evidence from the Late Cretaceous of Madagascar. Science 279: 1915-1919.

29. Perle A, Norell MA, Clark LM (1999) A new maniraptoran theropod—*Achillobator giganticus* (Dromaeosauridae)—from the Upper Cretaceous of Burkhant, Mongolia. Contrib Dept Geol Natl Univ Mong 101: 1-105.

30. Ostrom JH (1969) Osteology of *Deinonychus antirrhopus*, an unusual theropod dinosaur from the Lower Cretaceous of Montana. Bull Peabody Mus Nat Hist 30:1-165.

31. Wellnhofer P (1974) Das fünfte Skelettexemplar von *Archaeopteryx*. Palaeontogr Abt A 147: 169-216.

32. Czerkas SA, Yuan C (2002) An arboreal maniraptoran from northeast China. In: Cerkas SJ, editor. Feathered dinosaurs and the origin of flight. Blanding, Utah: The Dinosaur Museum. p. 63-95.

33. Norell MA, Makovicky PJ (1999) Important features of the dromaeosaurid skeleton II: information from newly collected specimens of *Velociraptor mongoliensis*. Am Mus Novit 3282: 1-45.

34. Zheng X, Xu X, You H, Zhao X, Dong Z (2009) A short-armed dromaeosaurid from the Jehol Group of China with implications for early dromaeosaurid evolution. Proc Roy Soc B 277:211-217.

35. Dames W, Kayser E (1884) Ueber Archaeopteryx. Palaeontol Abh 2: 119-196.

36. Mayr G, Pohl B, Harman S, Peters DS (2007) The tenth skeletal specimen of *Archaeopteryx*. Zool J Linn Soc 149: 97-116.

37. Wellnhofer P (1993) Das siebte Exemplar von *Archaeopteryx* aus den Solnhofener Schichten. Archaeopteryx 11: 1-47.

38. Xu X, Wang X, Wu X (1999) A dromaeosaurid dinosaur with filamentous integument from the Yixian Formation of China. Nature 401:262-266.

39. Xu X, Zhou Z, Wang X, Kuang X, Zhang F, Du X (2003) Four-winged dinosaurs from China. Nature 421: 335-340.

40. Turner AH, Pol D, Clarke JA, Erickson GM, Norell MA (2007). A basal dromaeosaurid and size evolution preceding avian flight. Science 317: 1378-1381.

41. Paul GS (1988) Predatory dinosaurs of the world. New York: Simon and Schuster.

42. Novas FE, Pol D (2005) New evidence on deinonychosaurian dinosaurs from the Late Cretaceous of Patagonia. Nature 433: 858-861.

43. Middleton KM (2001) The morphological basis of hallucal orientation in extant birds. J Morphol 250: 51-60.

44. Russell DA, Dong Z (1993) The affinities of a new theropod from the Alxa Desert, Inner Mongolia, People’s Republic of China. Can J Ea Sci 9: 375-402.
